# VPA mediates bidirectional regulation of cell cycle progression through the PPP2R2A-Chk1 signaling axis in response to HU

**DOI:** 10.1038/s41419-023-05649-8

**Published:** 2023-02-13

**Authors:** Benyu Su, David Lim, Chenyang Qi, Zhongwei Zhang, Junxiao Wang, Fengmei Zhang, Chao Dong, Zhihui Feng

**Affiliations:** 1 Department of Occupational and Environmental Health, School of Public Health, Cheeloo College of Medicine, Shandong University, Jinan, Shandong China; 2 Translational Health Research Institute, School of Health Sciences, Western Sydney University, Campbelltown, NSW Australia; 3 College of Medicine and Public Health, Flinders University, Bedford Park, SA Australia

**Keywords:** Checkpoint signalling, Prognostic markers, Stalled forks

## Abstract

Cell cycle checkpoint kinases play a pivotal role in protecting against replicative stress. In this study, valproic acid (VPA), a histone deacetylase inhibitor (HDACi), was found to promote breast cancer MCF-7 cells to traverse into G2/M phase for catastrophic injury by promoting PPP2R2A (the B-regulatory subunit of Phosphatase PP2A) to facilitate the dephosphorylation of Chk1 at Ser317 and Ser345. By contrast, VPA protected normal 16HBE cells from HU toxicity through decreasing PPP2R2A expression and increasing Chk1 phosphorylation. The effect of VPA on PPP2R2A was at the post-transcription level through HDAC1/2. The in vitro results were affirmed in vivo. Patients with lower PPP2R2A expression and higher pChk1 expression showed significantly worse survival. PPP2R2A D197 and N181 are essential for PPP2R2A-Chk1 signaling and VPA-mediated bidirectional effect on augmenting HU-induced tumor cell death and protecting normal cells.

## Introduction

Breast cancer is the most common malignancy in women worldwide and is curable in 70–80 % of patients with early-stage, non-metastatic disease [1]. Chemotherapy is a mainstay of breast cancer management, increasing patients’ long-term survival and decreasing their mortality [2, 3]; however, chemoresistance and adverse effects are significant causes of treatment failure [4, 5]. Locating an effective sensitizer has attracted much clinical interest lately [6].

Hydroxyurea (HU) depletes dNTPs to induce stalled or collapsed replication forks and is a commonly used antitumor drug with a known toxicology profile [7, 8]. Our previous study showed that tumor cells treated with 2 mM HU for 18 h resulted in one-end DNA double-strand breaks, activated the checkpoint kinase-1 (Chk1) signaling pathway and arrested tumor cells in the S phase [9, 10]. Chk1 coordinates the response to DNA damage [11–13] and is activated through its phosphorylation by the ataxia telangiectasia and RAD3-related (ATR) protein kinase [10, 14]. Chk1 signaling promotes cell cycle arrest and prevents the entry of cells with damaged or incompletely replicated DNA into mitosis, especially in cells lacking checkpoints in the cell cycle G1 phase [13, 15, 16]. The eukaryotic cell cycle is driven by the activation of cyclin-dependent kinases (CDKs) [17, 18]. The WEE1 kinase phosphorylates CDK1 and CDK2 on Tyr15 (Y15) to prevent progression to G2/M and S phases, respectively [18, 19].

Dephosphorylation is the most direct way to inactivate checkpoint kinases [20]. Phosphatase 2A (PP2A) is responsible for the most phosphatase activity in eukaryotic cells [21, 22]. PP2A is a heterotrimeric enzyme composed of scaffold subunit A (PP2A-A), regulatory subunit B (PP2A-B) and catalytic subunit C (PP2A-C) [23]. Numerous essential cellular functions, such as cell cycle, development, metabolism, and apoptosis, have been demonstrated to be regulated by PP2A [20, 23, 24]. The most prevalent and widely expressed regulatory subunit of PP2A is PPP2R2A (also known as B55α), which consists of seven-bladed β-propellers, with each of the blades comprising WD40 repeats [25, 26]. All known PPP2R2A-dependent substrates, such as P107 and c-Myc in cancer, are shown to be dysregulated and have critical roles in cell proliferation, differentiation, and survival [15, 26, 27]. A recent study showed that PPP2R2A deficiency makes non-small cell lung cancer cells more sensitive to Chk1 inhibition [15]. Whether and how PPP2R2A identifies Chk1 and affects its dephosphorylation, and whether explicitly targeting PPP2R2A to regulate Chk1 dephosphorylation remains unknown.

The histone deacetylase inhibitor (HDACi) family comprise four classes: I, IIa/IIb, III and IV. Some HDACis are approved in cancer treatment [28–31]. In our previous study, VPA, a class I and II HDACi, has been shown to increase the sensitivity of tumor cells to HU treatment; for example, VPA reduced S-phase arrest of the cell cycle and thus has a chemo-sensitizing role in response to HU-induced replication arrest by inhibiting Chk1 signaling [10, 32, 33]. The precise molecular mechanisms by which VPA sensitizes tumor cells through regulating checkpoint kinases in the various stages of the cell cycle and whether VPA affords protection on normal and abscopal cells to avoid HU toxicity remains elusive.

Here, we report that VPA has a bidirectional effect on the tumor and normal cell survival through selective regulation of cell cycle progression through the PPP2R2A-Chk1 pathway. PPP2R2A D197 and N181 play a critical role in regulating Chk1 dephosphorylation and VPA-mediated effects in response to HU treatment. In addition, the survival analysis showed that breast cancer patients with lower PPP2R2A expression and higher pChk1-S317 expression had significantly worse survival. This newly discovered mechanism of how VPA’s action is a step forward in understanding the plausible role of VPA as an inexpensive, efficacious adjuvant therapy in breast cancer management.

## Materials and methods

### Cell culture

MCF-7 and HEK293T cells were purchased from American Type Culture Collection (ATCC) and cultured as previously described [32]. 16HBE cell line was purchased from Stem Cell Bank, Chinese Academy of Sciences and maintained in DMEM medium with 10% fetal bovine serum (FBS). MCF-10A cell line was obtained from Stem Cell Bank, Chinese Academy of Sciences, cultured with Dulbecco’s Modified Eagle’s Medium/Nutrient Mixture F-12 Ham (Sigma-Aldrich, D9785) combined with 5% horse serum (Gibco, 26050088), 100 ng/ml Cholera toxin (Sigma-Aldrich, C8052), 20 ng/ml epidermal growth factor (Sigma-Aldrich, E5036), 0.5 μg/ml hydrocortisone (Sigma-Aldrich, 614157), 10 μg/ml human insulin (Sigma-Aldrich, I9278), and 1% penicillin–streptomycin (Sigma-Aldrich, V900929).

### Therapeutic agents

VPA (Sigma-Aldrich, P4543-100G) and HU (Sigma-Aldrich, H8627-25G) were purchased from Sigma-Aldrich. Chk1 inhibitor (LY2603618, Selleckchem, S2626) and PP2A inhibitor (LB-100, Selleckchem, S7537) were purchased from Selleck Chemicals.

### Plasmids

Plasmids used in this study were listed in the Supplementary Table S1.

### Antibodies

Antibodies used for Western blotting: anti-Chk1 (Santa Cruz, sc-8408, 1:500), anti-phospho-Chk1 S317 (CST, 2344, 1:1000), anti-phospho-Chk1 S345 (CST, 2348, 1:1000), anti-PP2A (Abcam, ab32104, 1:5000), anti-PPP2R2A (CST, 5689S, 1:1000), anti-WEE1 (Santa Cruz, sc-5285, 1:500), anti-p-Tyr15-cdc2 (CDK1) (CST, 4539, 1:1000), anti-pTBK1 (CST, S172, 1:1000), anti-TBK1 (Thermo Fisher Scientific, 67211-1-Ig, 1:2000), anti-c-Myc (ACE, AB001-03, 1:2000), anti-Flag (ACE, AB002-03, 1:2000), anti-HA (ACE, AB004-03, 1:2000), anti-phospho-Ser/Thr-Pro (Sigma-Aldrich, 05-368, 1:5000), anti-HDAC1 (Abcam, ab109411, 1:1000), anti-HDAC2 (Abcam, ab32117, 1:1000), anti-acetyl-Histone3 (Abcam, ab52946; 1:2000), anti-GAPDH (Zsbio, TA-08, 1:2000), Secondary antibodies included the goat anti-rabbit IgG-horseradish peroxidase conjugated (Pierce, 31460) and goat anti-mouse IgG-horseradish peroxidase conjugated IgG (Pierce, 31430).

Antibodies used for immunofluorescence (IF) staining experimentations: anti-phospho-Chk1 S317 (CST, 2344, 1:150), anti-p-Tyr15-cdc2 (CST, 4539, 1:50), anti-phospho-RPA2 Ser4/Ser8 (Thermo Fisher Scientific, A300-245A, 1:500), anti-γH2AX Ser139 (Sigma-Aldrich, 05-636, 1:500), AlexaFluor 594-labeled goat anti-mouse IgG (Thermo Fisher Scientific, A11032), AlexaFluor 488-labeled chicken anti-rat IgG (Thermo Fisher Scientific, A21470), and AlexaFluor 488-labeled chicken anti-rabbit IgG (Thermo Fisher Scientific, A11008).

### Treatment of cells

Cells were pre-treated with 0.5 mM VPA for 24 h before the addition of 2 mM HU for another 18 h. Similarly, cells were pre-treated with 1.5 μM LY2603618 or 2.5 μM LB-100 for 48 h alone or in combination with VPA or/and HU before subject to further experimental analysis.

### Treatment of animals

The female Sprague-Dawley (SD) rats used in this study were obtained from Jinan Peng Yue Experimental Animal Breeding Co., Ltd (Jinan, China). All animal experimental procedures were approved by the Shandong University Human and Animal Ethics Research Committee (81472800, approved March 2014). The 2,2’-Bis (hydroxymethyl) butyric acid (DMBA, Sigma, D3254) was dissolved in refined corn oil at a concentration of 20 mg/ml A single dose of 1-ml DMBA-oil solution was administered intragastrically (i.g) to SD rats. During the experiment, breast palpation was performed on rats 3 times a week to check for tumor, and the body weight was measured weekly. Primary tumors were found around the breast about 50 days after i.g DMBA administration. The rats were randomly divided into untreated, VPA, HU, and VPA + HU groups. The investigator was blinded to the group. The untreated group animals were given 0.9% saline. The VPA groups were treated with 200 mg/kg VPA intraperitoneal (i.p.) injection, once a day for 10 days. The HU groups were treated with 400 mg/kg HU (i.p.), once a day for 10 days. For the VPA + HU group, 4 h after administrating VPA (200 mg/kg), 400 mg/kg HU was administered i.p. once a day for 10 days. Rats received a 100 mg/kg of BrdU (i.p.) 24 h prior to tissue collection. The rats were euthanized humanely on the 66th day following the termination of the VPA and/or HU treatments in concordance with the animal ethics committee’s approval.

### siRNAs and transfections

Knockdown of PPP2R2A in MCF-7 or 16HBE cells was performed by transfecting 100 pmol siPPP2R2A or corresponding amounts of non-targeting control siRNA (Genepharma) with Opti-MEM and Lipofectamine2000 (Thermo Fisher Scientific, 12566014). For the combined knockdown of all two HDACs, 50 pmol siHDAC1 and 50 pmol siHDAC2 were combined to have pooled siRNAs. After 24–48 h, the transfection mixture was removed, and cells were stimulated with VPA and/or HU. Efficient knockdown was confirmed by Western blotting. Sequences of siRNAs used in this study are listed in Supplementary Table S2.

### Lentiviral particle packaging and transduction

To generate lentiviral particles, HEK293T cells were transiently co-transfected with the lentiviral-based expression target plasmids, psPAX2, and pMD2.G in a 4:3:1 ratio using polyethyleneimine (PEI) (Polysciences, 23966). Supernatants containing lentiviruses were filtered 48 h after transfection with an Acrodisc 25-mm syringe filter with a 0.45-μm membrane and utilized for cell transduction in the presence of 8 μg/ml polybrene (Solarbio, H8761).

### Generation of PPP2R2A-knockout (KO) cells using the CRISPR/Cas9 method

The gene-targeted PPP2R2A gRNAs were subcloned into LentiCRISPR V2 vector (Addgene, 52961) following standard procedures. In the presence of polybrene (Solarbio, H8761), virus particles containing PPP2R2A gRNAs were applied to MCF-7 cells twice at 24 h intervals. The transfected MCF-7 cells were selected in DMEM media containing 1 μg/ml puromycin (Sigma-Aldrich, P4512) for 1 week. KO cells were validated by Western blotting. Sequences of gRNAs are listed in Supplementary Table S3.

### RNA isolation and real-time qPCR

RNA extraction was performed with FastPure Cell/Tissue Total RNA Isolation Kit (Vazyme, RC101-01). 0.5 μg RNA was processed for reverse transcription using Hifair V one-step RT-gDNA digestion SuperMix (YESEN, 11142ES60). The real-time PCR was performed with the diluted cDNA, the indicated primers and Maxima SYBR Green qPCR Master Mix (Selleck, B21203) on Light Cycler® 480II (Roche Applied Science, Indianapolis, IN, USA). Data were acquired by Light Cycler® 480 Software and analyzed by 2−△△Ct method with GAPDH used as the internal reference gene. Primers used for RT-qPCR are listed in Supplementary Table S4.

### Western blot and co-immunoprecipitation

Cells were harvested and lysed with NETN buffer (20 mM Tris·HCl, pH 8.0, 100 mM NaCl, 0.5% Nonidet P-40, and 1 mM ethylenediaminetetraacetic acid [EDTA]) supplemented with benzonase nuclease (Millipore, E1014), protease inhibitor and phosphatase inhibitors for 30 min on ice. Whole-cell lysates were heated in sodium dodecyl sulfate (SDS) loading buffer, following resolution on polyacrylamide gel electrophoresis (PAGE), transfer to polyvinylidene fluoride (PVDF) membranes, and immunoblotting with the designated antibodies. For co-immunoprecipitation, whole-cell extracts were centrifuged at 15,000 rpm for 15 min at 4 °C. After the lysis, supernatants were incubated with 20 μl beads (Thermo Fisher Scientific, 88837) for 5 h, or Protein A beads (Santa Cruz, sc-2003) overnight at 4 °C with gentle rotation. Immunoblotting was performed after three ice-cold NETN buffer washes on protein-bound beads.

### Immunofluorescence

After treatment, cells were fixed with 3% paraformaldehyde (PFA) for 30 min and permeabilized with 0.2% Triton X-100 for 30 s at room temperature (RT). After washing twice with PBS, cells were blocked with 3% FBS before incubating with primary antibodies overnight at 4 °C. Secondary antibodies Alexa Fluor 488 (Thermo Fisher Scientific, A11008) and Alexa Fluor 594 (Thermo Fisher Scientific, A11032) were introduced to the cells at room temperature for an additional 60 min before staining with DAPI. The images from the immunofluorescence assays were viewed at ×100 magnification with a fluorescence microscope (Zeiss Axio Observer, Axio Observer7).

### DNA fiber assay

Exponentially growing cells were pulse-labeled with 25 μM 5-Iodo-2-deoxycytidine (IdU, Sigma-Aldrich, I7125) for 30 min before the end of the respective experimental treatment. After removing IdU, a second pulse labeling with 250 μM 5-chloro-2-deoxycytidine (CldU, Sigma-Aldrich, C6891) for 30 min was performed. The cells were harvested, lysed, and DNA fiber spreads were prepared. Immunofluorescence staining: fiber spreads were incubated with mouse anti-BrdU (BD, 347580) that recognizes IdU and subsequently rat anti-BrdU (Abcam, ab6326) against CldU. AlexaFluor 594-labeled goat anti-mouse IgG (Thermo Fisher Scientific, A11032) and AlexaFluor 488-labeled chicken anti-rat IgG (Thermo Fisher Scientific, A21470) were used to detect the primary antibodies. The images from the immunofluorescence assays were viewed at ×100 magnification with a fluorescence microscope (Zeiss Axio Observer, Axio Observer7). We employed ImageJ (1.8.0) to quantify the length of DNA spreads in order to calculate the replication fork speed (1μm corresponds to 2.59 kb) [34].

### Cycloheximide chase experiment

Cells were seeded onto 60-mm dishes with cell culture media supplemented with 50 μg/ml cycloheximide. Cells were harvested at indicated time points. Gel-Pro 32 analyzer software was used to quantify the relative expression level compared with control treatment.

### Denaturing immunoprecipitation

HEK293T cells transiently co-transfected with HA epitope-tagged Chk1 (Gift from Xingzhi, Xu [35]) with Flag-tagged vector or Flag epitope-tagged PPP2R2A were lysed with denaturing buffer (20 mM Tris·HCl, pH 8.0, 50 mM NaCl, 0.5% Nonidet P-40, 0.5% SDS, 0.5% deoxycholate, and 1 mM EDTA) on ice for 15 min and subsequently boiled at 95 °C for 5 min. The cell lysates were cooled down on ice for 5 min and incubated with Anti-HA magnetic beads (Thermo Fisher Scientific, 88837) for 3 h at 4 °C with gentle rotation. Before undergoing immunoblotting, protein-bound beads were washed with ice-cold denaturing buffer three times and boiled with SDS/PAGE sample-loading buffer.

### CCK8

Cells were seeded in 96-well plates at a density of 2 × 10^3^ cells per well. Following treatments, CCK8 solution (APE×BIO, Houston, USA) was added to the treated cells and incubated for 4 h at 37 °C. The absorbance of the solution was measured using an enzyme immunoassay analyzer (TECAN, Infinite M2000 PRO) at 450 nm.

### Clonogenic survival assay

The clonogenic survival assay method was described elsewhere [36, 37]. The number of cell colonies (≥50 cells per clone) was counted and cell survival was presented as the cell survival fraction (SF), with SF = (the number of clones/seeded cells)/plating efficiency (PE).

### Cell cycle analysis

10 μM of 5-Bromo-2-deoxyUridine (BrdU) (Sigma-Aldrich, B5002) was added to the cells 30 min before the end of treatment, and the collected cells were fixed in 70% ethanol overnight at −20 °C. After the cells were incubated with 0.4 mg/ml pepsin in 2 M HCl for 30 min, they were neutralized with 0.1 M sodium borate for 15 min. The cells were further incubated with the primary antibody of anti-BrdU (BD, 347580), and then incubated with a secondary antibody of AlexaFluor 594-labeled goat anti-mouse IgG (Thermo Fisher Scientific, A-11005). The nucleus was stained with 4′,6-diamidino-2-phenylindole (DAPI) for cell cycle analysis by flow cytometry (BD, ZE5).

### Histopathological analysis

The tissues were fixed in 4% paraformaldehyde solution for 48 h, then embedded in paraffin and sectioned (5 µm). Paraffin-embedded tissue sections were deparaffinized in xylene and rehydrated in ethanol solution. Sections were counterstained with hematoxylin and eosin (HE). The sections were scanned by a Pannoramic DESK scanner and viewed by CaseViewer 2.4 software.

### Immunohistochemistry analysis

Paraffin-embedded sections were deparaffinized and rehydrated as described above. Heat-induced epitope retrieval was performed, and endogenous peroxidase activity was inactivated by hydrogen peroxide. After the sections were incubated in goat serum, the slides were incubated in primary antibodies anti-BrdU (BD, 347580, 1:50), anti-53BP1 (Novus Biologicals, NB100-304, 1:1000), anti-phospho-Chk1 S317 (Bioworld, BS4040, 1:100), anti-phospho-RPA2 Ser4/Ser8 (Thermo Fisher Scientific, A300-245A, 1:1000), and anti-PPP2R2A (CST, 5689S, 1:100) overnight. The slides were then incubated with biotinylated secondary antibody (Citeab, BA-9200, 1:300) and (Citeab, BA-1000, 1:300), incubated with the ABC kit and diaminobenzidine (DAB), counterstained with hematoxylin [38]. The sections were scanned by a Pannoramic DESK scanner (3DHISTECH Ltd) and viewed by CaseViewer 2.4 software.

### Tissue array analysis

One hundred forty samples of cancerous tissues were purchased from Shanghai Outdo Biotech Company (Shanghai, China) per the ethics approval from Shanghai Outdo Biotech Company (YB M-05-01), and informed consent was signed by all the patients involved in this study. Immunohistochemistry staining of pChk1-S317 and PPP2R2A in tissues was performed, and the density (H-score) of pChk1-S317 and PPP2R2A expression in the tissues was calculated and analyzed by Student’s *t*-test. Breast cancer patient information is listed in Supplementary Tables S5–S7.

### Statistical analysis

Continuous values were expressed as mean ± standard deviation (SD). The unpaired two-tailed Student’s *t*-test was utilized to compare the groups. The log-rank test was used for the survival curves. Kaplan–Meier Plotter tools were used to analyze the correlation between the mRNA expression of PPP2R2A and Chk1, and the survival of patients with breast cancer. The GEPIA database was used to analyze the expression of PPP2R2A and Chk1 with tumor stage of breast cancer. All statistical analyses were performed using GraphPad Prism 9 (GraphPad Prism Software). Statistical significance is indicated as follows: ns, not significant; *P < 0.05, **P < 0.01.

## Results

### The bidirectional effects of VPA on **cell cycle progression** is dependent on Chk1 activation in response to HU treatment

**Fig. 1:**
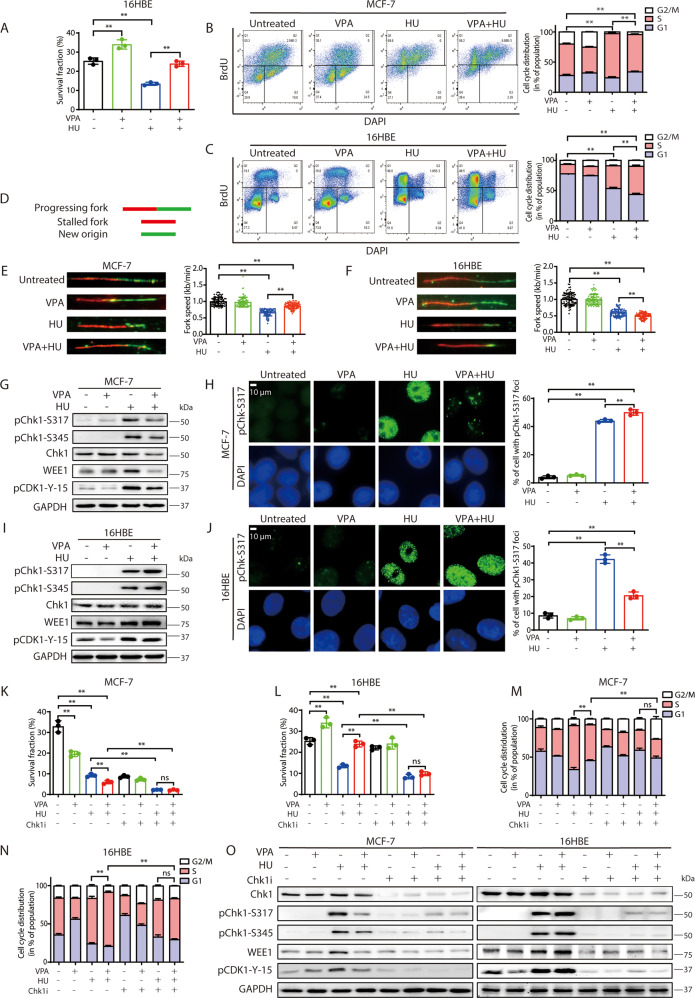
The bidirectional effects of VPA on cell cycle progression depend on Chk1 activation in response to HU treatment. **A** Survival fraction of 16HBE cells after exposure to 0.5 mM VPA and/or 2 mM hydroxyurea (HU) was detected by clonogenic assay. Quantification was from three independent experiments. **P < 0.01. **B**, **C** MCF-7 and 16HBE cells were treated with 0.5 mM VPA and/or 2 mM HU. Cell cycle analysis of the DNA content of MCF-7 and 16HBE cells was analyzed by flow cytometry. Cell cycle analysis is shown as mean ± SD (*n* = 3). Statistical significance is displayed for S cells. **P < 0.01. **D**–**F** DNA fiber assay: IdU (red tracks) and CldU (green tracks) were detected using specific primary and secondary antibodies. Replication tracks were classified according to schematic shown in (**D**). Representative images and quantitative analysis of replication tracks are shown (**E**) and (**F**). Data are presented as mean ± SD (*n* = 3). **P < 0.01. **G**, **I** MCF-7 and 16HBE cells were treated as stated in **A**. Whole-cell lysates were subjected to western blotting analysis and probed with pChk1-S317, pChk1-S345, Chk1, WEE1 and pCDK1-Y-15 antibodies. GAPDH was used as loading control. **H**, **J** MCF-7 and 16HBE cells were treated as described in **A**. After fixation, cells were counterstained with pChk1-S317 specific antibody (green). DAPI was used to visualize nuclei. Representative images are shown (scale bar, 10μm). Quantification of positive signal of pChk1-S317 was from three independent experiments. **P < 0.01. **K**, **L** MCF-7 and 16HBE cells were subjected to colony formation assay in the presence of 0.5 mM VPA, 2 mM HU or/and 1.5μM Chk1 inhibitor (Chk1i) LY2603618. Data are presented as mean ± SD (*n* = 3). **P < 0.01; ns, not significant. **M**, **N** MCF-7 and 16HBE cells were treated as stated in **K** and **L**. Cell cycle analysis of the DNA content of MCF-7 (**M**) and 16HBE (**N**) cells were analyzed by flow cytometry. Cell cycle analysis is shown as mean ± SD (*n* = 3). Statistical significance is displayed for S cells. **P < 0.01; ns, not significant. **O** MCF-7 and 16HBE cells were treated as described in **K** and **L**, whole-cell lysates were subjected to western blotting analysis. GAPDH was used as loading control.

We previously demonstrated that VPA inhibits cancer cell growth after HU treatment [10, 32]. In the current study, we found that normal 16HBE (Fig. 1A) and MCF-10A (Supplementary Fig. S1A) cells exhibited significantly increased proliferation following the combined VPA and HU treatment (VPA + HU) as compared with HU-alone (*P* < 0.01), suggesting that VPA has a bidirectional effect on attenuating cancer cells growth and promoting normal cells proliferation.

To investigate the differential effects of VPA on the cancer and normal cell cycle, we treated both cell lines with the therapeutic equivalent dose of 0.5 mM VPA. We found the VPA treatment promoted the HU-treated breast cancer MCF-7 cells to slide into the G2/M phase (Fig. 1B, *P* < 0.01). In the normal cell lines, the VPA + HU treatment arrested the 16HBE (Fig. 1C, *P* < 0.01) and MCF-10A (Supplementary Fig. S1B, *P* < 0.01) cells in the S phase and delayed their transition into G2/M phase. The DNA fiber assay measured replication fork dynamics (Fig. 1D). The VPA + HU-treated MCF-7 cells had incorporated more new origins and re-started DNA synthesis faster than HU-alone treated cells (Fig. 1E, *P* < 0.01). By comparison, the VPA + HU treated 16HBE cells had decreased formation of new origins with green labeled nucleotides and re-started DNA synthesis was also reduced (Fig. 1F, *P* < 0.01).

Since activated checkpoint kinases of G2/M phase play a pivotal role in controlling cell cycle progression, we next investigated whether VPA regulates the checkpoint kinases. VPA + HU treatment significantly decreased the levels of pChk1-S317, pChk1-S345, Chk1, WEE1 and pCDK1-Y-15 in MCF-7 cells (Fig. 1G). In addition, from the immunofluorescence staining, VPA treatment diminished the pChk1-S317 and pCDK1-Y-15 foci formation induced by HU in the MCF-7 cells (Fig. 1H and Supplementary Fig. S1C), and increased the levels of checkpoint kinases in the 16HBE and MCF-10A cells (Fig. 1I and Supplementary Fig. S1D), and HU-induced pChk1-S317 foci formation in 16HBE cells (Fig. 1J, *P* < 0.01). These data alluded to a potential VPA’s bidirectional regulation of Chk1-mediated checkpoint kinases at G2/M phase for tumor and normal cells.

To determine whether Chk1 participates in the observed VPA-bidirectional effects, we pharmacologically inhibited Chk1 using LY2603618. We found the previously observed bidirectional regulation effect of VPA is significantly abolished in cell survival fraction (Fig. 1K, L, ns), cell cycle profiling in the S phase (Fig. 1M, N, Supplementary Fig. S1E, F, ns) and the checkpoint kinase Chk1, pChk1-S317, pChk1-S345, WEE1 and pCDK1-Y-15 levels (Fig. 1O), indicating that the VPA-bidirectional effects are Chk1 dependent.

We also ascertained the formation of DNA double-strand breaks (DSBs) and the ability of cells to repair single-stranded DNA (ssDNA) to assess the effect of VPA on replication fork stability. We found the foci formation of phosphorylated histone H2AX (γH2AX) as a DSBs marker was significantly increased in the MCF-7 cells treated by VPA + HU as compared with HU-alone. The foci formation of replication protein A 2 phosphorylation (pRPA2 at Ser4 and/or Ser8), as a ssDNA repair marker, showed the opposite changes (Supplementary Fig. S2A, *P* < 0.01). In contrast, the γH2AX foci formation was significantly decreased in the 16HBE cells treated by VPA + HU as compared to HU-alone, and the pRPA2 foci formation also showed similar changes (Supplementary Fig. S2B, *P* < 0.01). The results indicated that the observed VPA-bidirectional effects on tumor and normal cells involved regulation of replication fork stability. Importantly, after Chk1 inhibition, the VPA-bidirectional effects to regulate replication fork stability was significantly abolished in both tumor and normal cells (Supplementary Fig. S2A and B, ns), suggesting that the VPA-bidirectional effects are Chk1 dependent.

### PP2A is involved in VPA-bidirectional effects

**Fig. 2:**
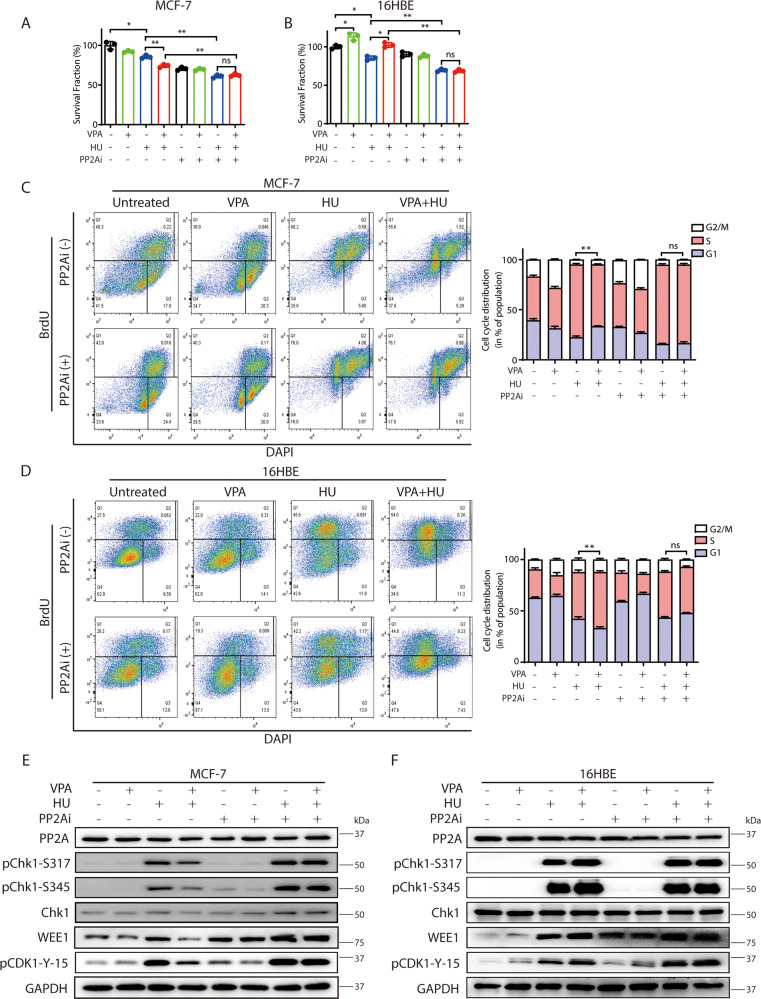
Phosphatase PP2A participates in VPA-mediated bidirectional regulation under HU treatment. **A**, **B** Survival fraction of MCF-7 and 16HBE cells after exposure to 0.5 mM VPA, 2 mM HU or/and 2.5 μM PP2A inhibitor (PP2Ai) LB-100 were detected by CCK8 assay. Quantification was from three independent experiments. *P < 0.05, **P < 0.01; ns, not significant. **C**, **D** MCF-7 and 16HBE cells were treated as stated in **A** and **B**. Cell cycle analysis of the DNA content of MCF-7 and 16HBE cells were analyzed by flow cytometry. Cell cycle analysis is shown as mean ± SD (*n* = 3). Statistical significance is displayed for S cells. **P < 0.01; ns, not significant. **E**, **F** MCF-7 and 16HBE cells were treated as described in **A** and **B**, whole-cell lysates were subjected to Western blotting analysis. GAPDH was used as loading control.

It was previously reported that ATR could directly phosphorylate Chk1 for the regulation of G2/M phase checkpoint [39], however, it is unclear how Chk1 dephosphorylation is regulated. As PP2A dephosphorylates checkpoint kinases such as ATM and Chk2 are involved in Chk1 dephosphorylation [20], we next investigate if VPA might regulate cell cycle and Chk1 dephosphorylation through PP2A. We employed the PP2A inhibitor LB-100 to test this hypothesis. We found that LB-100 treatment significantly enhanced the phosphorylation of the known substrate TBK1 [40] suggesting LB-100 worked in our study system (Supplementary Fig. S3A). The inhibition of PP2A in both MCF-7 and 16HBE cells exhibiting the previously observed VPA-bidirectional effects resulted in a significant abolition in cell survival (Fig. 2A, B, ns), cell cycle progression through the S phase (Fig. 2C, D, ns), and Chk1 phosphorylation at both S317 and S345 (Fig. 2E, F). Importantly, we noticed that PP2A inhibition significantly increased Chk1 phosphorylation at both Ser317 and Ser345 in response to HU treatment, but did not affect the PP2A protein levels in our working system (Fig. 2E, F). These data suggest that PP2A is involved in the regulation of Chk1 dephosphorylation and VPA-bidirectional effects on cell cycle and cell survival.

### PP2A regulatory subunit PPP2R2A contributes to VPA-bidirectional effects through Chk1-associated pathway

**Fig. 3:**
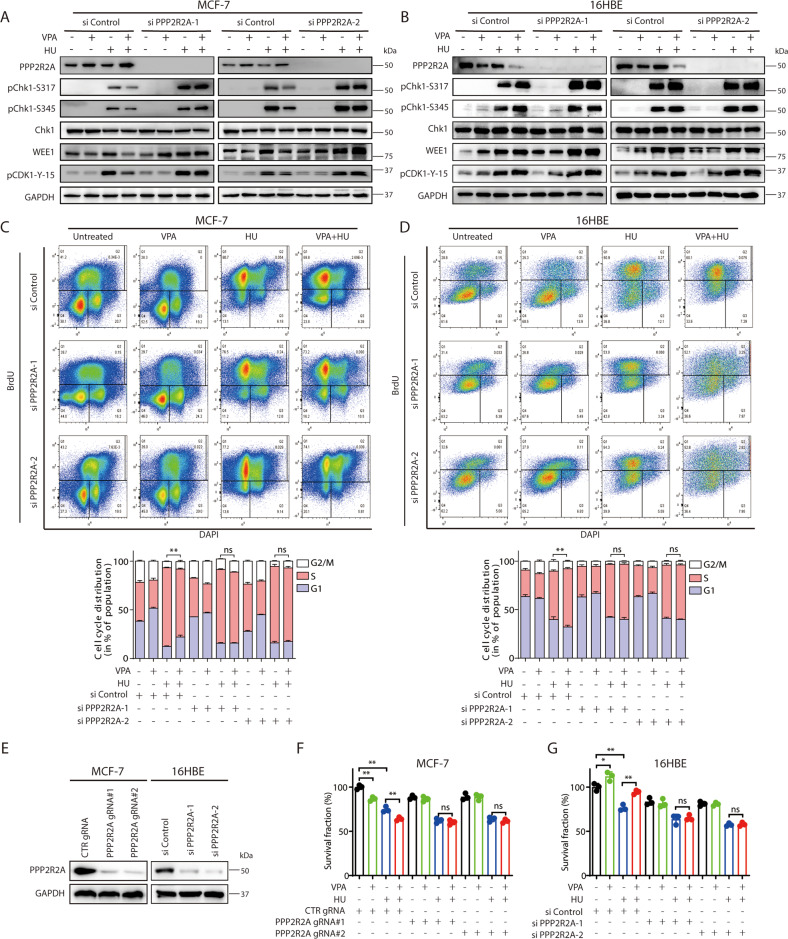
PPP2R2A regulates the cell survival of cancer and normal cells through Chk1-associated pathway in response to HU treatment. **A**, **B** MCF-7 and 16HBE cells were transiently transfected with indicated PPP2R2A-targetiing siRNAs (siPPP2R2A-1 and siPPP2R2A-2). After 24 h, cells were treated with 0.5 mM VPA and/or 2 mM HU. The protein levels of PPP2R2A, pChk1-S317, pChk1-S345, Chk1, WEE1 and pCDK1-Y-15 were analyzed by Western blotting. GAPDH was used as loading control. **C**, **D** MCF-7 and 16HBE cells were treated as stated in **A** and **B**. Cell cycle analysis of the DNA content of MCF-7 and 16HBE cells were performed by flow cytometry. Cell cycle analysis is shown as mean ± SD (*n* = 3). Statistical significance is displayed for S cells. **P < 0.01; ns, not significant. **E**–**G** MCF-7 cells were transduced with non-targeting gRNA (CTR gRNA) or PPP2R2A-targeting gRNAs (PPP2R2A gRNA#1 and PPP2R2A gRNA#2), and 16HBE cells were transiently transfected with indicated siPPP2R2A (siPPP2R2A-1 and siPPP2R2A-2). Western blotting was performed to evaluate PPP2R2A expression (**E**). Cell survival in either MCF-7 cells (**F**) or 16HBE cells (**G**) treated by 0.5 mM VPA or/and 2 mM HU was detected by CCK8 assay. Each data point in the graph was from three independent experiments, *P < 0.05, **P < 0.01; ns not significant.

Since PP2A is involved in the observed VPA-bidirectional effects, we next investigate which subunit of PP2A may be involved. A recent study reported an association between PP2A B-regulatory subunit (PPP2R2A) deficiency and Chk1 inhibition in non-small cell lung cancer [15]. Thus, we speculate that PPP2R2A may dephosphorylate Chk1. Interestingly, we found the protein expression of PPP2R2A was increased in MCF-7 cells treated with VPA-alone or VPA + HU, correlated with reduced Chk1 phosphorylation at Ser317 and Ser345 (Fig. 3A). In contrast, the PPP2R2A protein expression decreased with VPA-alone or VPA + HU in 16HBE cells (Fig. 3B). In knocking out the PPP2R2A gene expression in the MCF-7 and 16HBE cells, the previously observed VPA-bidirectional effects were significantly eliminated, specifically in the phosphorylation of Chk1 (at S317 and S345) and CDK1 (pCDK1-Y-15) (Fig. 3A and Fig. 3B), and cell cycle progression through the S phase (Fig. 3C, D, ns). Also, PPP2R2A-depleted MCF-7 cells (by its gRNA) or PPP2R2A-knockdown 16HBE cells (by its siRNA) exhibited significant reduction in the previously observed VPA-bidirectional effects (Fig. 3E–G and Supplementary Fig. S4A and B, ns). The above data are consistent with the proposition that VPA regulates Chk1 dephosphorylation through PPP2R2A.

### Examination of VPA-bidirectional effects in vivo

**Fig. 4:**
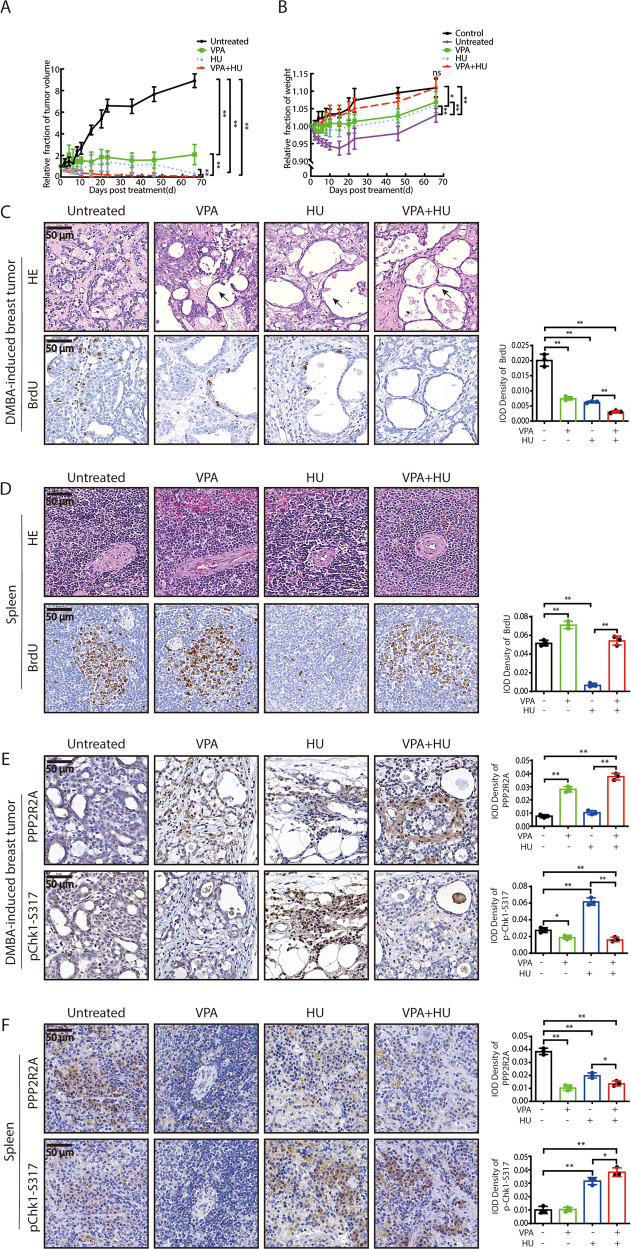
Bidirectional regulation of VPA on PPP2R2A and checkpoint kinases under HU treatment in vivo. **A** The rats with breast tumor induced by DMBA were randomly divided into untreated (*n* = 3), 200 mg/kg VPA (*n* = 3), 400 mg/kg HU (*n* = 3), and VPA + HU (*n* = 3) groups. The dynamic change of rat tumor volume in untreated, VPA, HU, and VPA + HU groups is shown. **P < 0.01. **B** The graph shows the dynamic change of relative weight of rat breast tumor. The rats with breast tumor induced by DMBA were treated as described in **A**. Normal rat was used as Control. **P < 0.01; ns, not significant. **C**, **D** The rats were treated as described in **A**. The morphological change of tumor and spleen in groups after treatment, the expression of BrdU by immunohistochemistry staining in tumor and spleen tissues were showed. The arrows indicate necrotic cells. Quantification was from three independent experiments. **P < 0.01. **E**, **F** The rats were treated as depicted in **A**. Representative images shows the protein expression of PPP2R2A and pChk1-S317 assessed by immunohistochemistry staining in tumor and spleen tissues, respectively. Notes: integrated optical density (IOD) of indicated proteins in immunohistochemistry images was quantified. Each data point in the graph was from three independent experiments, *P < 0.05, **P < 0.01.

We next used a primary rat model of DMBA-induced breast cancer that we had described previously [10, 32] to triangulate the in vitro data. In this animal model, we concurred with previous studies that VPA + HU treatment produced a significant reduction in tumor volume (Fig. 4A, *P* < 0.01), there were also decreased cell proliferation indicated by BrdU labeling in (Fig. 4C, *P* < 0.01). During the course of observation, the VPA + HU treatment recovered the decrease in rat body weight induced by HU- or VPA-alone treatments, there was no significant difference in the body weight between the control and VPA + HU groups at the endpoint (Fig. 4B, ns), indicating that VPA not only enhanced HU sensitivity to tumor tissues but also minimized HU-induced toxicity to normal tissues in the rats. Based on this and previously reported study [41], we sampled the spleen for further in vivo validation of the earlier observed findings. Although there was no abnormal morphological change in the spleen among the treatment groups by HE staining, VPA mitigated against the decreased cell proliferation ability induced by HU (Fig. 4D, *P* < 0.01), consistent with earlier findings.

We found the level of PPP2R2A was lower in rat breast tumor than in the spleen tissues, and the level of pChk1-S317 was higher in the breast tumor than in the spleen tissues (Supplementary Fig. S5A, B, *P* < 0.01). We observed the expression of PPP2R2A and pChk1-S317 in serial sections by IHC staining, the combination of VPA and HU significantly enhanced the PPP2R2A level but decreased the pChk1-S317 level in breast tumor tissues (Fig. 4E, *P* < 0.01). The VPA + HU treatment decreased the PPP2R2A level but enhanced the pChk1-S317 level in the spleen tissues (Fig. 4F, *P* < 0.01). Together, these data suggest there is a negative correlation between PPP2R2A and pChk1-S317, and VPA may exert bidirectional regulation through PPP2R2A-Chk1 signaling axis under HU treatment in vivo, which is consistent the in vitro results.

### PPP2R2A promotes the dephosphorylation of Chk1 at Ser317 and Ser345 with HU treatment

**Fig. 5:**
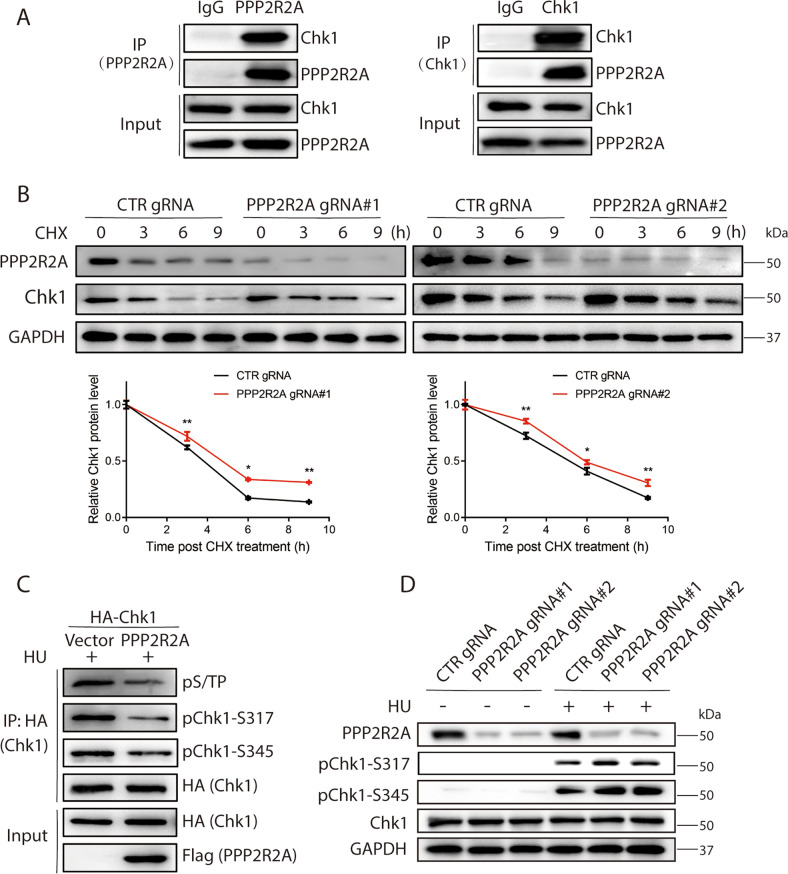
PPP2R2A promotes the dephosphorylation of Chk1 at Ser317 and Ser345 under HU treatment. **A** Reciprocal co-immunoprecipitation analysis between PPP2R2A and Chk1. MCF-7 cells were lysed and processed for co-immunoprecipitation and Western blotting experimentations using the indicated antibodies. **B** PPP2R2A deficiency stabilizes Chk1 protein level. MCF-7 cells with or without PPP2R2A knockout with two independent gRNAs were treated with cycloheximide (50 μg/ml) and were subjected to Western blotting analysis at the indicated time points. The protein expression of Chk1 was quantified from three independent experiments. *P < 0.05, **P < 0.01. **C** 293T cells transiently co-transfected with Flag-PPP2R2A and HA-Chk1 were lysed and immunoprecipitated by anti-HA antibody under denaturing condition. Western blotting analysis was performed using the indicated antibodies. **D** MCF-7 cells transduced with two independent PPP2R2A-targeting gRNAs were treated with 2 mM HU for 18 h. Whole-cell lysates were subjected to Western blotting and probed with indicated antibodies. GAPDH was used as loading control.

To investigate the association between PPP2R2A and Chk1 phosphorylation, we first examined PPP2R2A and Chk1 protein interaction by co-immunoprecipitation, and found the endogenous PPP2R2A interacts with Chk1 (Fig. 5A). Next, we investigate the effect of PPP2R2A on Chk1 protein stability. The protein turnover of Chk1 was measured by cycloheximide (CHX) chase assay and we found slower degradation of Chk1 in CRISPR/Cas9-mediated PPP2R2A-KO cells as compared to control cells, indicating Chk1 is stabilized in the absence of PPP2R2A (Fig. 5B, *P* < 0.01). Consistent results were also obtained in the siRNA-mediated PPP2R2A-silencing cells (Supplementary Fig. S6A, *P* < 0.01). Next, we performed denaturing immunoprecipitation and found PPP2R2A promotes the dephosphorylation of pChk1-S317 and pChk1-S345 with HU treatment (Fig. 5C). The PPP2R2A-mediated Chk1 dephosphorylation was also verified in the PPP2R2A-knockout cells (Fig. 5D). The above data indicate that PPP2R2A interacts with Chk1 to regulate its stability for promoting the dephosphorylation of Chk1 at Ser317 and Ser345.

### PPP2R2A D197 and N181 are essential for Chk1 dephosphorylation and VPA-mediated bidirectional regulation in response to HU treatment

**Fig. 6:**
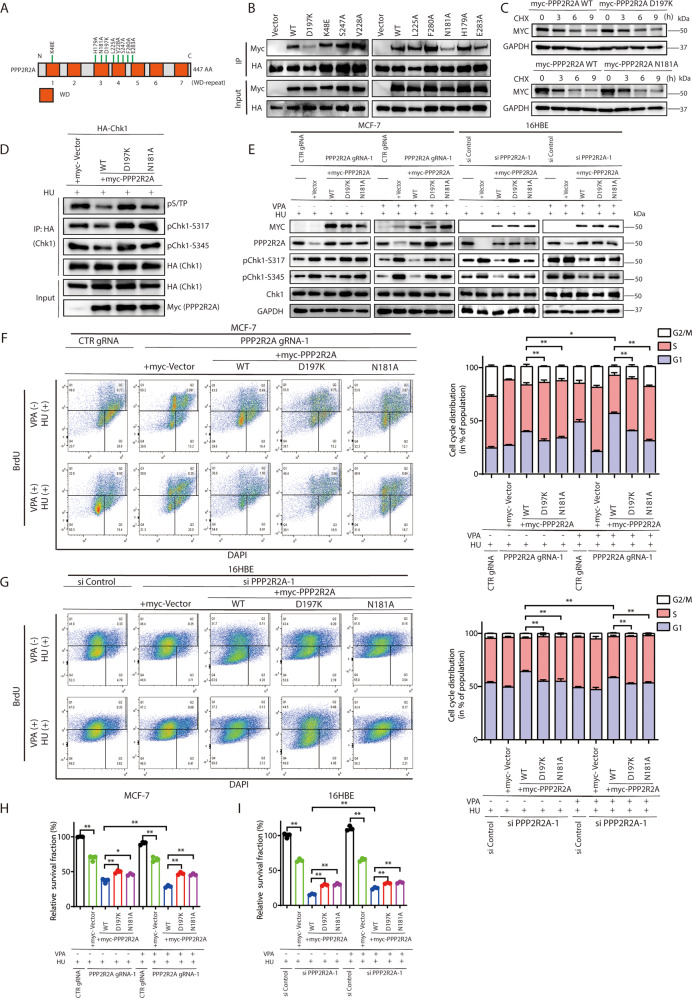
PPP2R2A D197 and N181 are essential for Chk1 dephosphorylation and VPA-mediated bidirectional regulation in response to HU treatment. **A** The schematic representation of the residues in top acidic face of PPP2R2A used in this study. **B** Representative immunoprecipitation experiment to test Chk1 binding requirements on Myc-PPP2R2A. HA-Chk1 and and Myc-PPP2R2A (wild-type, WT or mutant) constructs were co-transfected into 293T cells and subjected to immunoprecipitation. These assays were resolved via SDS-PAGE, and proteins were detected using anti-HA and anti-Myc antibodies. **C** MCF-7 cells transiently transfected with Myc-PPP2R2A WT, Myc-PPP2R2A D197K and Myc-PPP2R2A N181A were treated with cycloheximide (50 μg/ml). Whole-cell lysates were subjected to western blotting at the indicated time points and probed with Myc-PPP2R2A antibody. GAPDH was used as loading control. **D** 293T cells transiently co-transfected HA-Chk1, co-with Myc-Vector, Myc-PPP2R2A WT, Myc-PPP2R2A D197K and Myc-PPP2R2A N181A were lysed and immunoprecipitated with anti-HA antibody under denaturing condition. Western blotting analysis was performed using pS/TP, Myc-PPP2R2A, HA-Chk1, pChk1-S317 and pChk1-S345 antibodies. **E** MCF-7 and 16HBE cells pretreated with the indicated gRNAs or siRNA were transfected with Myc-Vector or Myc-tagged PPP2R2A WT, D197K and N181A mutants. The cells were treated with 0.5 mM VPA and/or 2 mM HU, Western blotting experiments were performed to evaluate the level of Myc-PPP2R2A, PPP2R2A, pChk1-S317, pChk1-S345 and Chk1. GAPDH was used as loading control. **F**, **G** MCF-7 and 16HBE cells were treated as described in **E**. Cell cycle analysis of the DNA content of cells were performed by flow cytometry. Cell cycle analysis is shown as mean ± SD (*n* = 3). Statistical significance is displayed for S cells. *P < 0.05, **P < 0.01. **H**, **I** MCF-7 and 16HBE cells were treated as stated in **E**. The survival of MCF-7 and 16HBE cells was detected by CCK8 assay. Each data point in the graph was from three independent experiments. *P < 0.05, **P < 0.01.

To further investigate which sites in PPP2R2A would be essential for the regulation of Chk1 dephosphorylation and contribute to VPA-bidirectional effects, we used 9 Myc-tagged PPP2R2A variants (Fig. 6A) which lie in the top of the β-propeller [42, 43]. By co-immunoprecipitation, we found PPP2R2A D197K and N181A variants showed the most profound impact on the binding of Chk1 when directly compared to wild-type (WT) PPP2R2A (Fig. 6B). However, both PPP2R2A D197K and N181A variants had no noticeable effect on the protein stability of PPP2R2A as tested by the CHX chase assay (Fig. 6C). In addition, using denaturing immunoprecipitation, we found the overexpression of PPP2R2A D197K and PPP2R2A N181A variants failed to reduce the phosphorylation of Chk1 at Ser317 and Ser345 as compared to PPP2R2A WT, suggesting that the amino acids of PPP2R2A D197 and N181 are essential for PPP2R2A-mediated Chk1 dephosphorylation (Fig. 6D). We then performed complementation experiments with PPP2R2A WT, D197K and N181A mutants in PPP2R2A knockout MCF-7 cells and PPP2R2A-knockout 16HBE cells (Fig. 6E). Notably, reconstitution of PPP2R2A-depleted MCF-7 and 16HBE cells with PPP2R2A WT alone decreased the phosphorylation of Chk1 both at Ser317 and Ser345 under HU treatment, but reconstitution with the PPP2R2A D197K or N181A mutant failed to dephosphorylate Chk1 (Fig. 6E), indicating that both sites of PPP2R2A D197 and N181 were required for the regulation of Chk1 dephosphorylation. Under HU and VPA combination treatment, the reconstitution with the PPP2R2A WT further decreased phosphorylation of Chk1 in MCF-7 cells compared with HU-alone treatment, there is an inverse increased phosphorylation of Chk1 compared to HU-alone treatment in 16HBE cells; but the reconstitution with the PPP2R2A D197K and N181A mutants failed to alter the phosphorylation of Chk1 (Fig. 6E), further indicating that both sites of PPP2R2A D197 or N181 were able to regulate VPA-bidirectional effects in response to HU treatment for Chk1 dephosphorylation. Moreover, after the reconstitution with the PPP2R2A D197K and N181A mutants, VPA-bidirectional effects for cell cycle progression (Fig. 6F, G, *P* < 0.01) and cell survival (Fig. 6H, I, *P* < 0.01) were also eliminated. Together, the above results suggest that PPP2R2A D197 and N181 are essential for the regulation of Chk1 dephosphorylation and VPA-mediated bidirectional effects in response to HU treatment.

### VPA bidirectionally regulates PPP2R2A at the post-transcriptional level but not at transcription level by inhibiting HDAC1/2

**Fig. 7:**
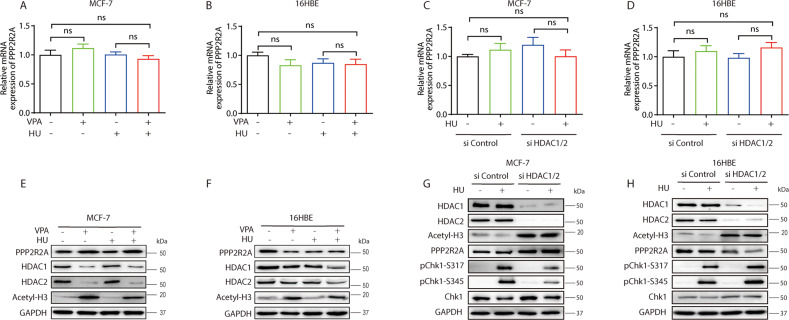
VPA bidirectionally regulates PPP2R2A at the post-transcriptional level by inhibiting HDAC1/2. **A**, **B** Relative mRNA levels of PPP2R2A in MCF-7 and 16HBE cells after exposure to 0.5 mM VPA and/or 2 mM HU was evaluated by quantitative RT-PCR. **C**, **D** MCF-7 and 16HBE cells transfected with two independent HDAC1 and HDAC2 targeting siRNAs were treated with 2 mM HU and cells were collected for quantitative RT-PCR. **E**, **F** MCF-7 and 16HBE cells were treated as stated in **A**. Whole-cell lysates were subjected to Western blotting analysis and probed with PPP2R2A, HDAC1, HDAC2 and Acetyl-H3 antibodies. GAPDH was used as loading control. **G**, **H** MCF-7 and 16HBE cells were transiently transfected with indicated HDAC1 and HDAC2 targeting siRNAs. After 24 h, cells were treated with 2 mM HU. The protein levels of HDAC1, HDAC2, Acetyl-H3, PPP2R2A, pChk1-S317, pChk1-S345 and Chk1 were analyzed by Western blotting. GAPDH was used as loading control, ns not significant.

Since this study demonstrated that VPA was able to regulate PPP2R2A function in both tumor and normal cells, so it would be necessary to further distinguish if VPA plays a role on PPP2R2A at the transcription or post-transcription level. To answer this question, we firstly detected PPP2R2A mRNA expression in both MCF-7 and 16HBE cells after VPA or/and HU treatment. The results indicated that MCF-7 and 16HBE cells exhibited no significant changes in PPP2R2A mRNA expression following VPA or/and HU treatment (Fig. 7A, B, ns). Since VPA is a specific inhibitor for HDAC1/2, so it would be essential to answer whether the effect of VPA on PPP2R2A was HDAC1/2-dependent at transcription level. After knocking down HDAC1/2 by its pooled siRNAs (Fig. 7G, H), PPP2R2A mRNA expression did not change significantly in both MCF-7 and 16HBE cells (Fig. 7C, D, ns), similar to the VPA effects. Secondly, we confirmed whether VPA-related PPP2R2A change was at the post-transcription level through HDAC1/2. Our results showed that VPA specifically inhibited HDAC1/2 expression by increasing the level of acetylated histone H3 in both MCF-7 and 16HBE cells in response to HU treatment (Fig. 7E, F). The elimination of HDAC1/2 by its siRNAs was also seen the increased in the level of acetylated histone H3, similar VPA’s effects after HU treatment in both cells (Fig. 7G, H). The levels of elevated PPP2R2A and decreased pChk1-S317/S345 were observed in MCF-7 cells (Fig. 7G), while the opposite was observed in the 16HBE cells (Fig. 7H). The above results support the proposition that the effect of VPA on PPP2R2A is at the post-transcription level through HDAC1/2 not at the transcription level.

### The PPP2R2A-Chk1 signaling axis correlates to the prognosis of patients with breast cancer

**Fig. 8:**
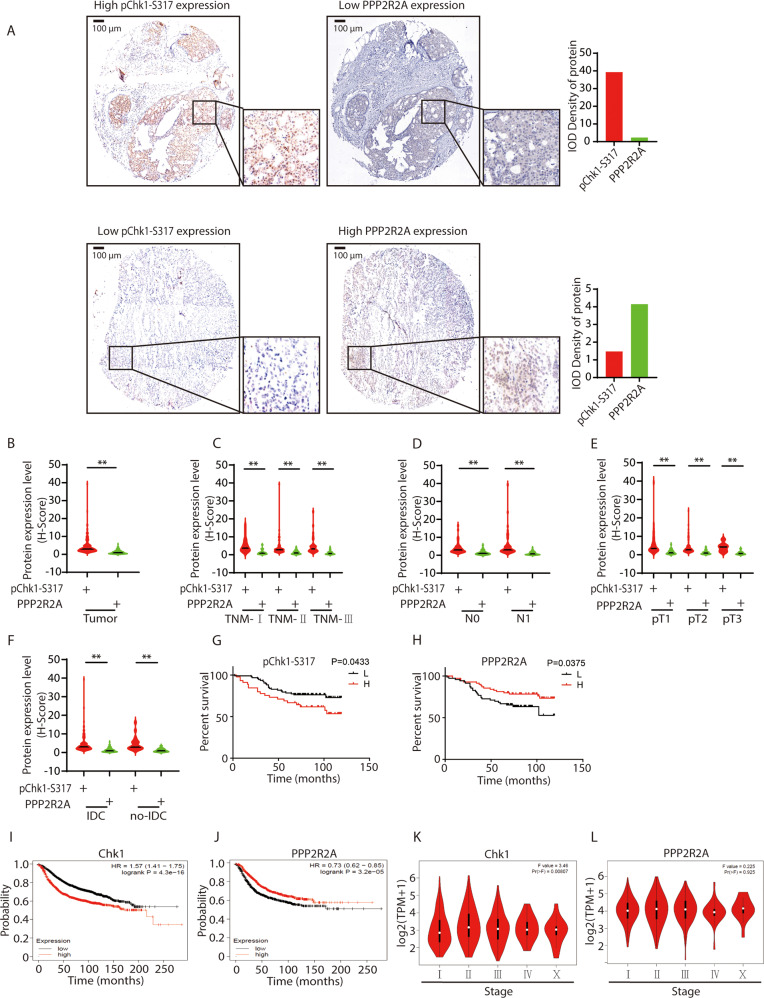
Association of the expression of PPP2R2A and pChk1 with the prognosis of patients with breast cancer. **A** Representative IHC images (magnification 10 and 50) of pChk1-S317 and PPP2R2A staining in a microarray consisting of 137 samples derived from breast cancer patients. Zoomed images show the details of the area denoted with a black square. Bar, 100 μm. Quantification of IOD density of pChk1-S317 and PPP2R2A from the representative IHC images. **B** PPP2R2A level negatively correlates with pChk1-S317 level in breast cancer patient tissues. H-Score is calculated using Aipathwell (*n* = 137). Student’s *t*-test. **P < 0.01. Tumor Node Metastasis (TNM) stage (**C**), lymph node metastasis (**D**), tumor size (**E**), and pathological type (**F**) had no effect on the expression of pChk1-S317 and PPP2R2A in tumor tissues. Quantification of pChk1-S317 and PPP2R2A in breast cancer patients with different TNM stages (TNM-I (*n* = 23), TNM-II (*n* = 76), TNM-III (*n* = 34)) (**C**), lymph node involvement (N0 (*n* = 43), N1 *(n* = 46)) (**D**), tumor size (pT1 (*n* = 38), pT2 (*n* = 84), pT3 (*n* = 13)) (**E**), Histological type (IDC: invasive ductal carcinoma (*n* = 118); No-IDC: non-invasive ductal carcinoma (*n* = 19)) (**F**) is shown. **P < 0.01. (**G**, **H**) High levels of pChk1-S317 or low levels of PPP2R2A predicate the worst prognosis in breast cancer patients. Survival analysis of patients with PPP2R2A/pChk1-S317 at high or low levels is shown (PPP2R2A: **L**: *n* = 69, **H**: *n* = 69; pChk1-S317: **L**: *n* = 93, **H**: *n* = 45). *P* < 0.05. **I**, **J** The prognostic value of mRNA levels of PPP2R2A and Chk1 in breast cancer patients was analyzed by Kaplan-Meier Plotter. *P* < 0.05. **K**, **L** The correlation between PPP2R2A and Chk1 expression and tumor stage in breast cancer patients was analyzed using the GEPIA database. **P < 0.01.

To assess the clinical relevance of the PPP2R2A-Chk1 signaling axis, we next performed IHC staining to examine PPP2R2A and pChk1-S317 in serial sections of human primary breast cancer specimens (n = 137) (Supplementary Figs. S7A and S8A). We found elevated expression of pChk1-S317 was accompanied by low expression of PPP2R2A, whereas increased PPP2R2A expression was associated by low pChk1-S317 expression (Fig. 8A). The analysis indicated a negative correlation between pChk1-S317 expression and PPP2R2A expression was also consistently observed in human primary breast tumor tissues (Fig. 8B, *P* < 0.01). This relationship was also consistent with the tumor node metastasis (TNM) stages, lymph node metastasis, tumor size, and pathological type (Fig. 8C–F, *P* < 0.01).

Next, the levels of pChk1-S317 and PPP2R2A derived from IHC staining analysis were divided into two categories: low-level group and high-level group. It is worth noting that breast cancer patients with high protein levels of pChk1-S317 or low levels of PPP2R2A show worse survival (Fig. 8G, H, *P* < 0.05). Further Kaplan-Meier Plotter tools were used to analyze the correlation between the mRNA expression of PPP2R2A and Chk1 and the survival of patients with breast cancer. Our analysis found that patients with decreased expression of PPP2R2A and increased expression of Chk1 had significantly worse survival (Figs. 8I, J, *P* < 0.05). We next analyzed the expression of PPP2R2A and Chk1 with tumor stage of breast cancer using GEPIA database, and found that Chk1 group varied significantly among tumor stage of breast cancer but not the PPP2R2A group (Fig. 8K, L). Taken together, these data reveal a negative correlation between PPP2R2A and pChk1-S317, highlighting the clinical relevance of PPP2R2A–Chk1 signaling axis in the progress of patients with breast cancer.

## Discussion

**Fig. 9:**
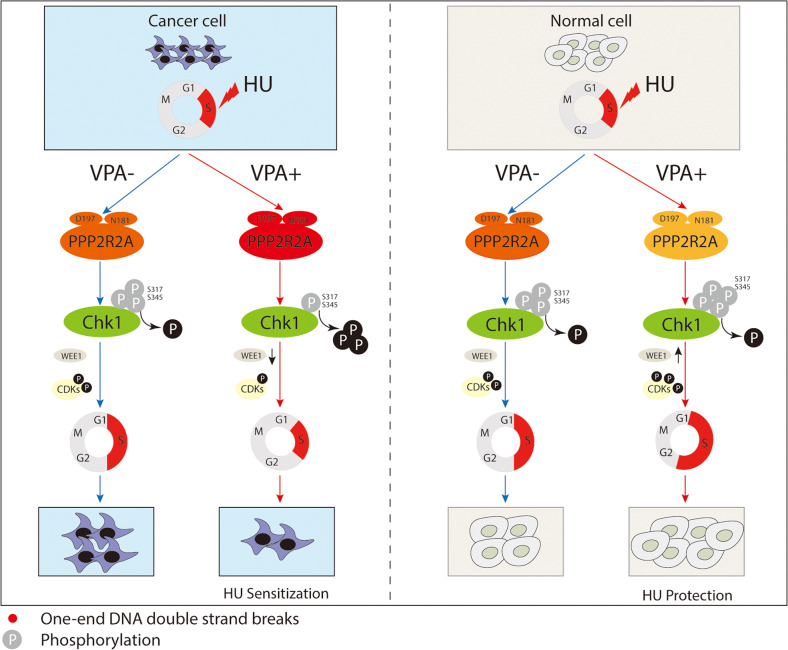
Schematic model. Schematic model illustrates VPA-mediated bidirectional regulation of cell cycle progressionthrough the PPP2R2A-Chk1 signaling axis in response to HU.

In this study, we demonstrate that the combined treatment of VPA and HU promotes the death of tumor tissue cells and protects normal tissue cells from chemotherapeutic agents both in vivo and in vitro. Our study reveals that the well-established HDAC inhibitor, VPA controls cell cycle progression by regulating PPP2R2A-Chk1 signaling axis in response to HU treatment. VPA reduced the phosphorylation of checkpoint kinases, allowed breast cancer cells to escape from HU-induced S phase block traversing into G2/M phase for catastrophic injury. Contrarily, VPA facilitated the phosphorylation of checkpoint kinases, arrested more normal cells in the S phase and delayed their transition into G2/M phase. Importantly, we found that PPP2R2A D197 and N181 amino acids are essential for the observed VPA-bidirectional effects on tumor and normal cells by regulating Chk1 dephosphorylation at Ser317 and Ser345, cell cycle control and cell survival in response to HU treatment. To our knowledge, this is the first study that shows that VPA exerts not only chemo-sensitizing effect on tumor cells but also a protective effect on normal cells by regulating cell cycle progression, supporting the proposition of trialing VPA as a low-cost, efficacious adjuvant therapy for breast cancer management (Fig. 9).

PP2A is a critical Ser/Thr protein phosphatase that relies on its B-regulatory subunits to specifically recruit substrates [42, 44, 45]. Previous reports have highlighted that PPP2R2A may perform different functions due to the potential presence of different substrate recruitment sites [46–48]. In our study, we found that the phosphorylation of Chk1 was regulated by PP2A (Figs. 2E, F), and further found that PPP2R2A and Chk1 were in the same protein complex (Fig. 5A). PPP2R2A affected the protein stability of Chk1 and promoted the dephosphorylation of Chk1 at Ser317 and Ser345 (Fig. 5B–D). We first identified the PPP2R2A-Chk1 signaling axis and demonstrated that VPA exerts the bidirectional regulatory effect through this signaling axis in response to HU treatment.

Recently, structural analysis of PPP2R2A revealed that the acidic top adjacent to the deep groove is thought to mediate its binding to various substrates [26]. We obtained 9 Myc-tagged PPP2R2A variants (D197K, K48E, S247A, V228A, L225A, F280A, N181A, H179A, and E283A), all of which are located in the highly conserved residues of the top of PPP2R2A. We showed that Chk1 binds to a PPP2R2A surface groove that is defined by residues PPP2R2A D197 and N181 (Fig. 6B), and PPP2R2A D197 and N181 play a critical role in Chk1 dephosphorylation at Ser317 and Ser345 (Figs. 6D–I). In addition, PPP2R2A D197 was previously reported to be a key site for Tau and P107 recruitment [42, 43], suggesting PPP2R2A D197 might serve as a conserved pivotal amino acid in mediating PPP2R2A substrate recognition. This acidic top may use substrate specific sequences to distinguish its different binding partners, and the PPP2R2A D197 or/and PPP2R2A N181 may be key recognition sites for multiple substrates. Importantly, we uncovered that PPP2R2A regulates cell cycle progression, cell survival and VPA-mediated bidirectional response to HU treatment through PPP2R2A D197 and N181 in both tumor and normal cells.

PPP2R2A appears to be a tumor suppressor gene that is frequently absent or under-expressed in most human tumors, such as prostate cancer, ovarian cancer, acute myelocytic leukemia (AML), and breast cancer [26, 49–53], pointing that PPP2R2A may play a role in tumorigenesis. In this study, we revealed the negative relationship of between PPP2R2A and pChk1-S317 is associated with the survival of breast cancer patients (Fig. 8G and H), potentially indicating the status of PPP2R2A or PPP2R2A-Chk1 pathway may also serve as a potential biomarker to predict the prognosis of tumor patients and guide tumor treatment. Interestingly, VPA not only has a chemo-sensitizing role on tumors, but it can also reduce the toxicity of HU on normal tissues through regulating PPP2R2A-Chk1, and reducing the damage of HU to normal tissue cells, strongly suggesting VPA would be an ideal chemo-sensitizer. VPA is currently used clinically as an anticonvulsant and mood-stabilizing medication to treat bipolar disorder and epilepsy [54], HU is a cancer chemotherapeutic agent used to treat head and neck cancer, brain tumors and melanoma [8], here we found the new efficacy of the combination of two drugs to breast cancer therapy, so our data provide important experimental and theoretical evidence for their clinical application.

We noticed a recent research report that HDAC1 and HDAC2 integrate checkpoint kinase phosphorylation through the phosphatase-2A subunit PR130 [20]. VPA is the inhibitor of HDAC1 and HDAC2, and regulates PPP2R2A protein levels, accompanied by increased acetylation of H3 (Figs. 7E, F). Importantly, the effect of VPA on PPP2R2A was at the post-transcription level through HDAC1/2 but not at the transcription level. It would be raised the question of how HDAC1/2 regulate the PPP2R2A function, and the interaction between both the proteins may be direct or indirect, which needs to be explored further.

In summary, we demonstrated that VPA exerts bidirectionally effect on regulating cell cycle progression through PPP2R2A (D197 and N181)-Chk1 signaling axis in tumor and normal cells. The findings provide novel insights into the molecular mechanisms of potential VPA + HU treatment in breast cancer, exemplifying a potential new therapeutic strategy for breast cancer management.

## Supplementary information

Supplementary Figure 1

Supplementary Figure 2

Supplementary Figure 3

Supplementary Figure 4

Supplementary Figure 5

Supplementary Figure 6

Supplementary Figure 7

Supplementary Figure 8

Supplementary Figure legend

Supplementary Table S1

Supplementary Table S2

Supplementary Table S3

Supplementary Table S4

Supplementary Table S5

Supplementary Table S6-pChk1-S317

Supplementary Table S7-PPP2R2A

Original western blots

Author Contribution Statement

Agreement from all authors

Change of authorship request form - Journals

## Data Availability

The datasets generated and/or analyzed during the current study are included within the article and are available from the corresponding authors upon reasonable request.
