# Supplementary figures and images for: VPA mediates bidirectional regulation of cell cycle progression through the PPP2R2A-Chk1 signaling axis in response to HU
Source: Cell Death Dis. 2023 Feb 13;14(2):114. doi: 10.1038/s41419-023-05649-8 (PMC9925808; doi:10.1038/s41419-023-05649-8)

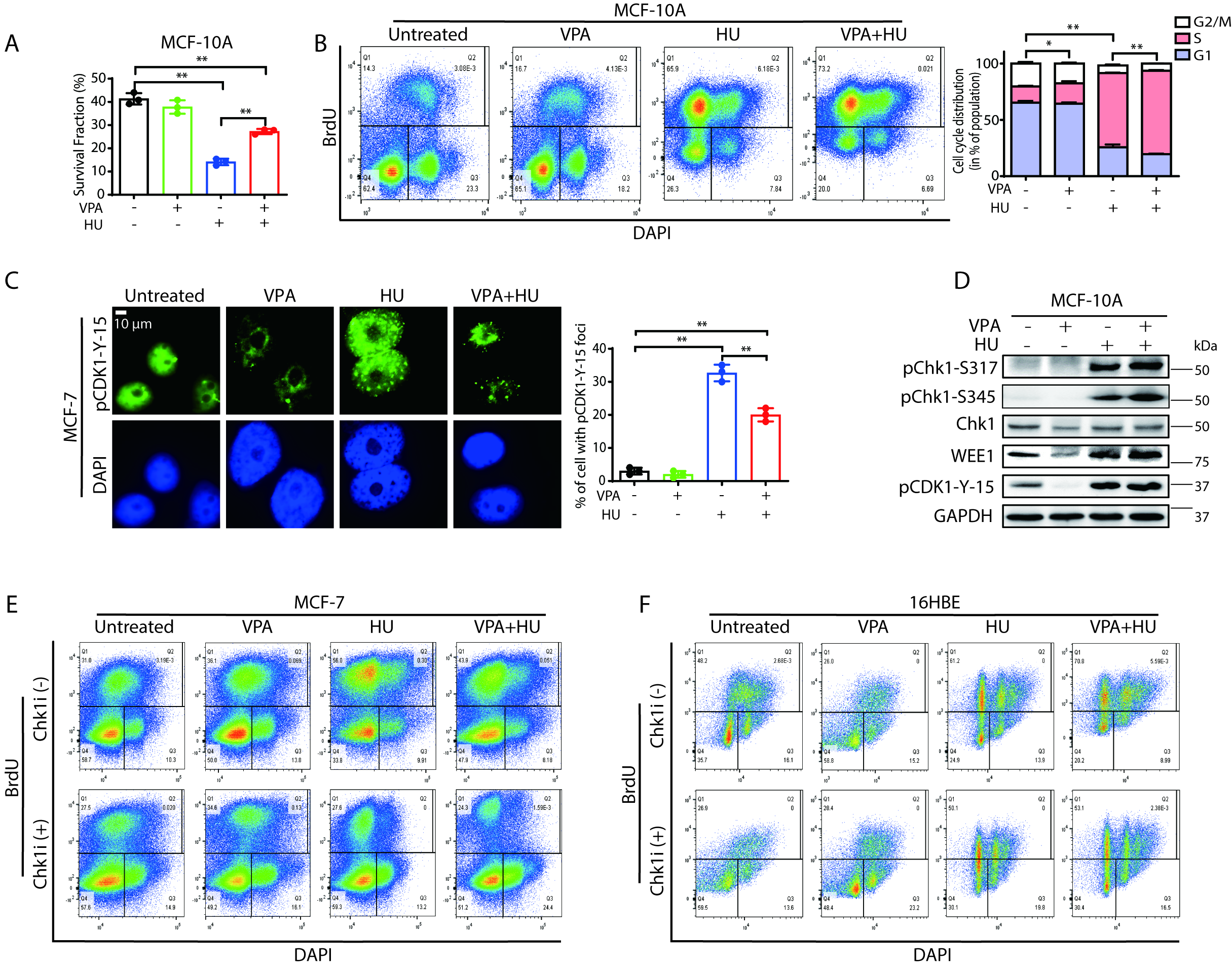

Supplement: Supplementary file 1 — Supplementary Figure 1 [file 41419_2023_5649_MOESM1_ESM.tif]

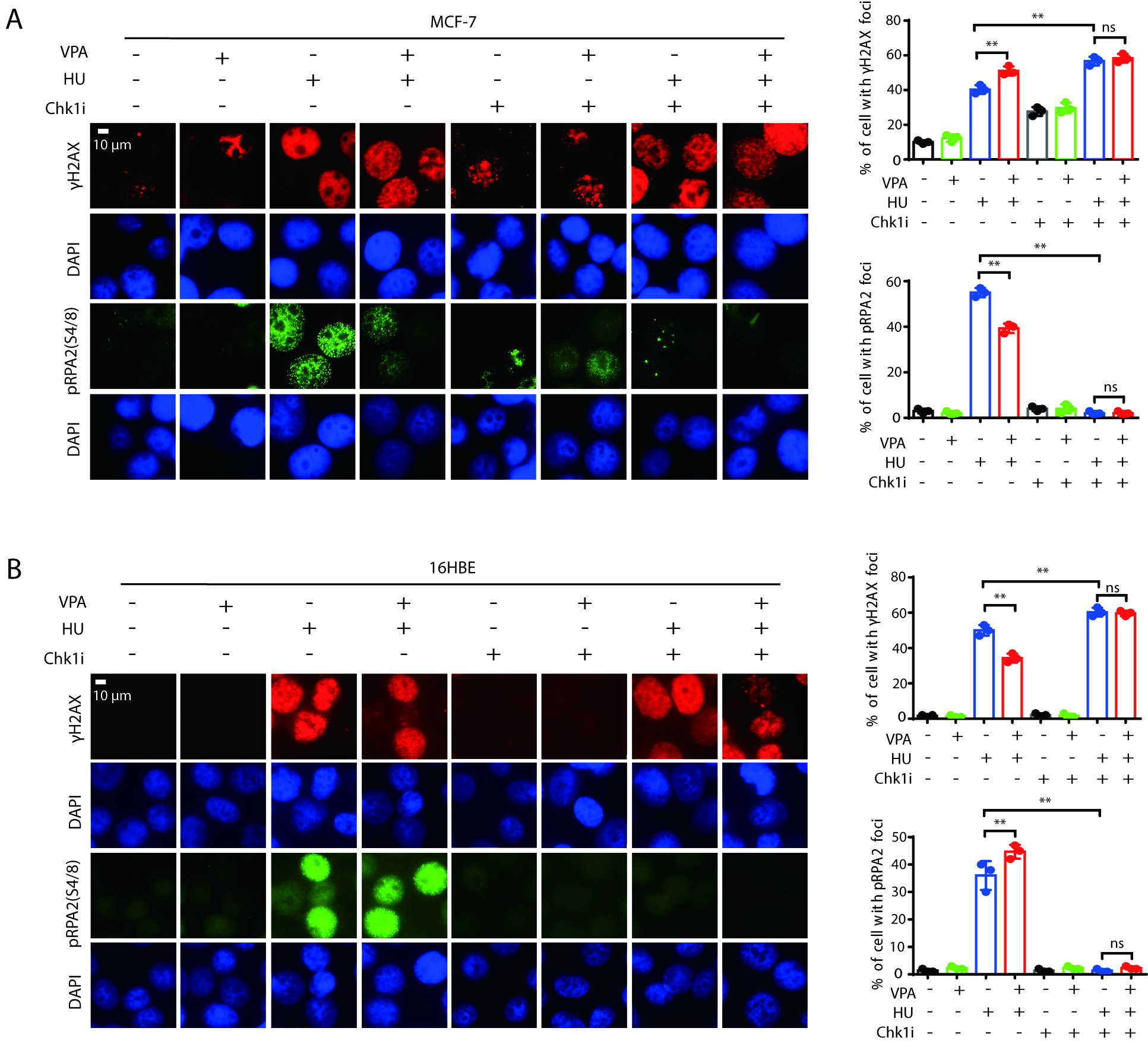

Supplement: Supplementary file 2 — Supplementary Figure 2 [file 41419_2023_5649_MOESM2_ESM.tif]

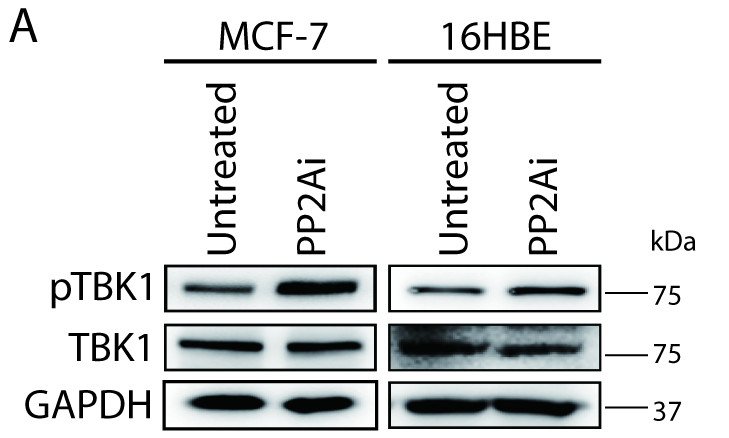

Supplement: Supplementary file 3 — Supplementary Figure 3 [file 41419_2023_5649_MOESM3_ESM.tif]

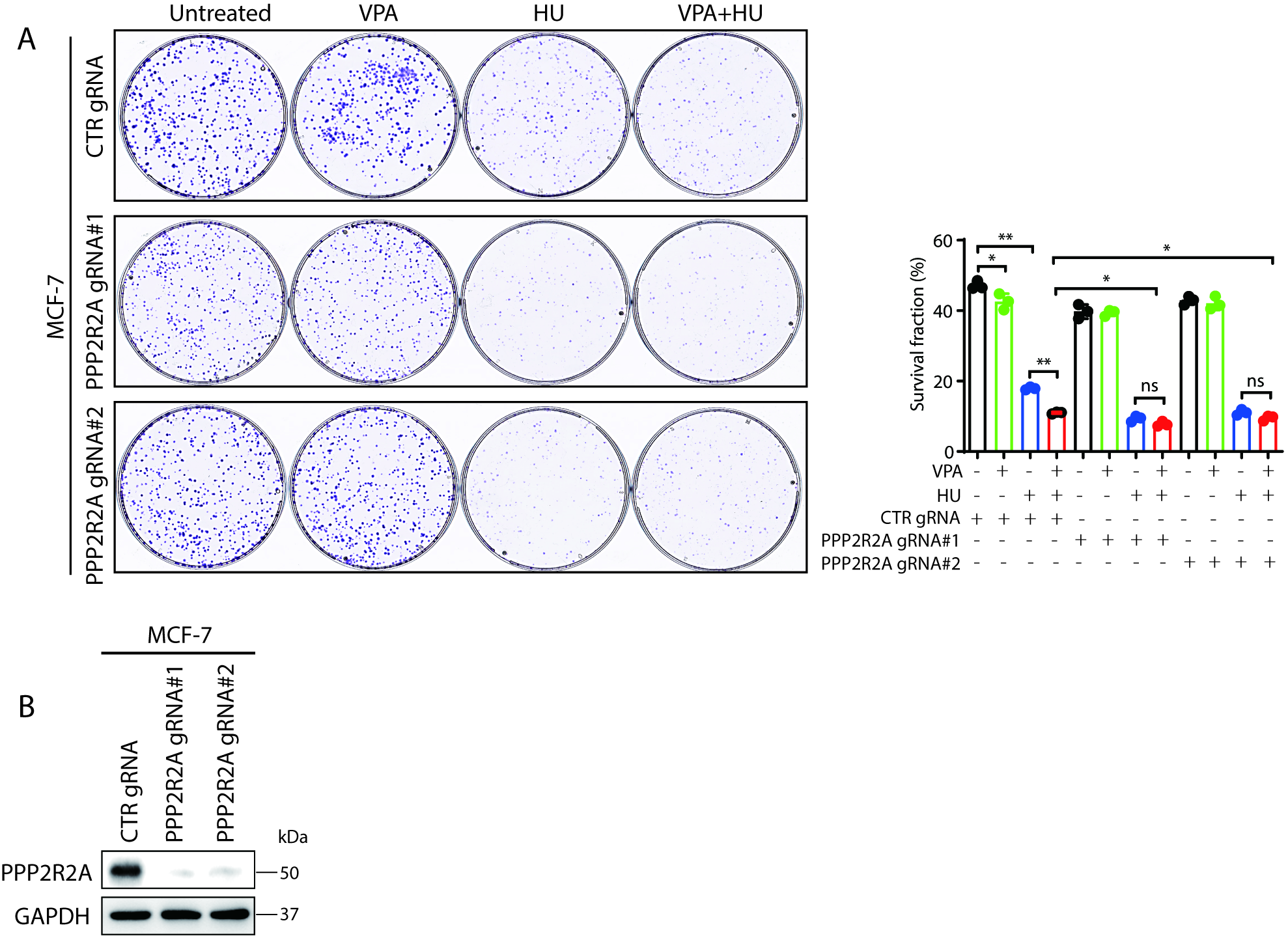

Supplement: Supplementary file 4 — Supplementary Figure 4 [file 41419_2023_5649_MOESM4_ESM.tif]

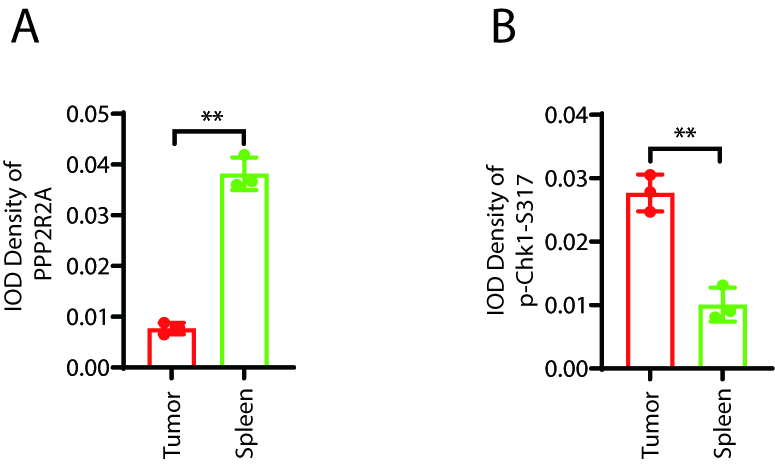

Supplement: Supplementary file 5 — Supplementary Figure 5 [file 41419_2023_5649_MOESM5_ESM.tif]

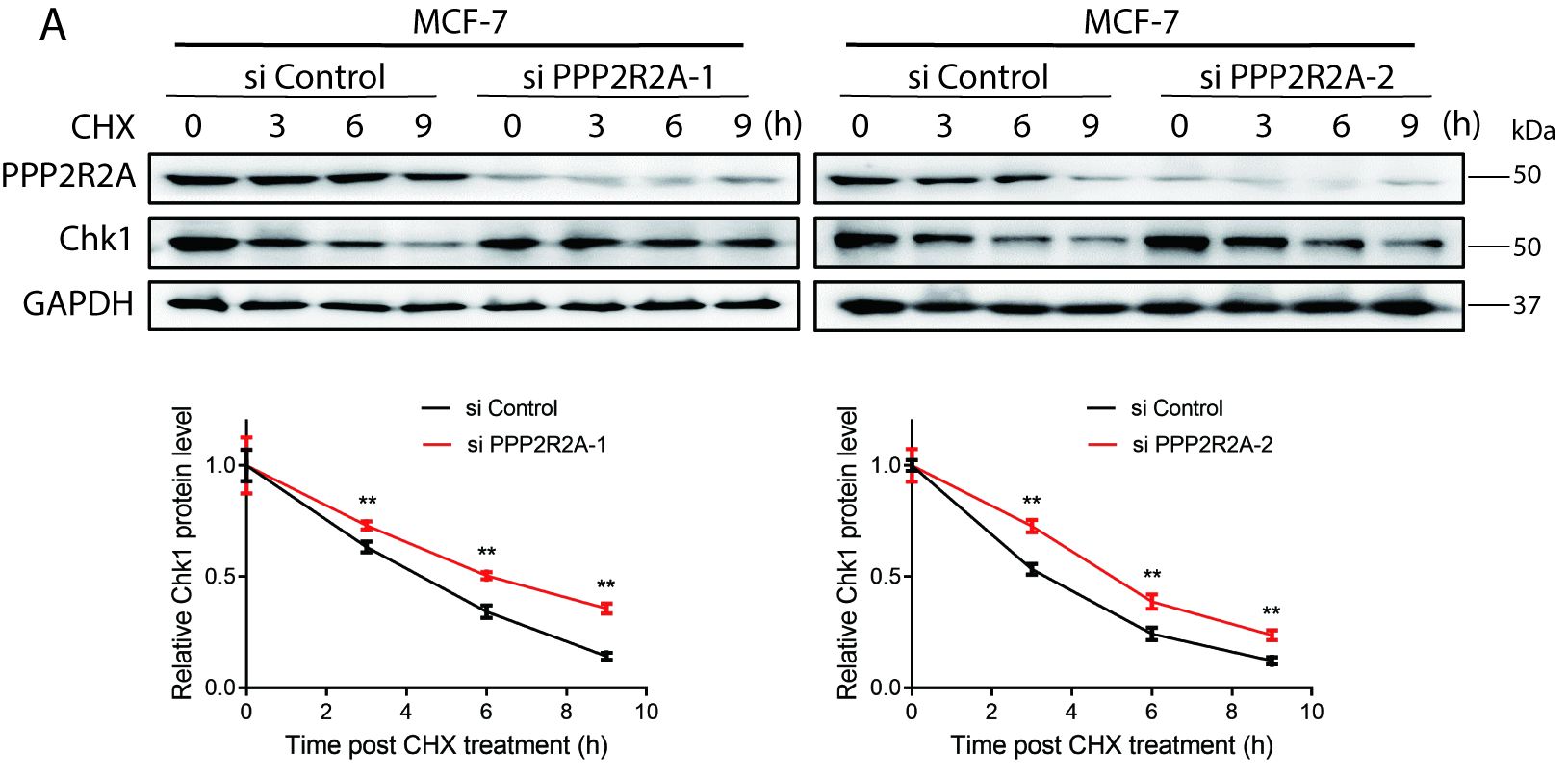

Supplement: Supplementary file 6 — Supplementary Figure 6 [file 41419_2023_5649_MOESM6_ESM.tif]

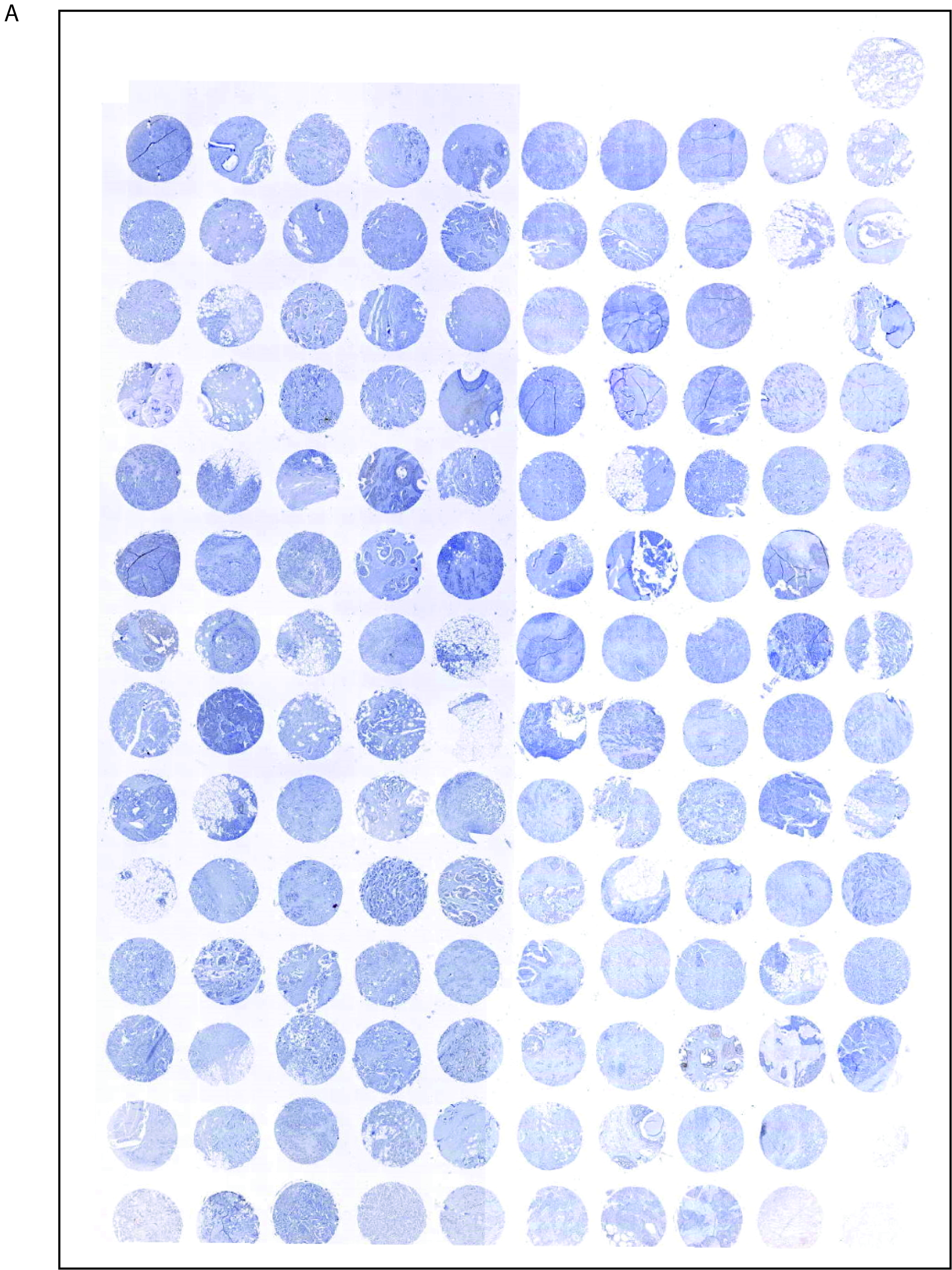

Supplement: Supplementary file 7 — Supplementary Figure 7 [file 41419_2023_5649_MOESM7_ESM.tif]

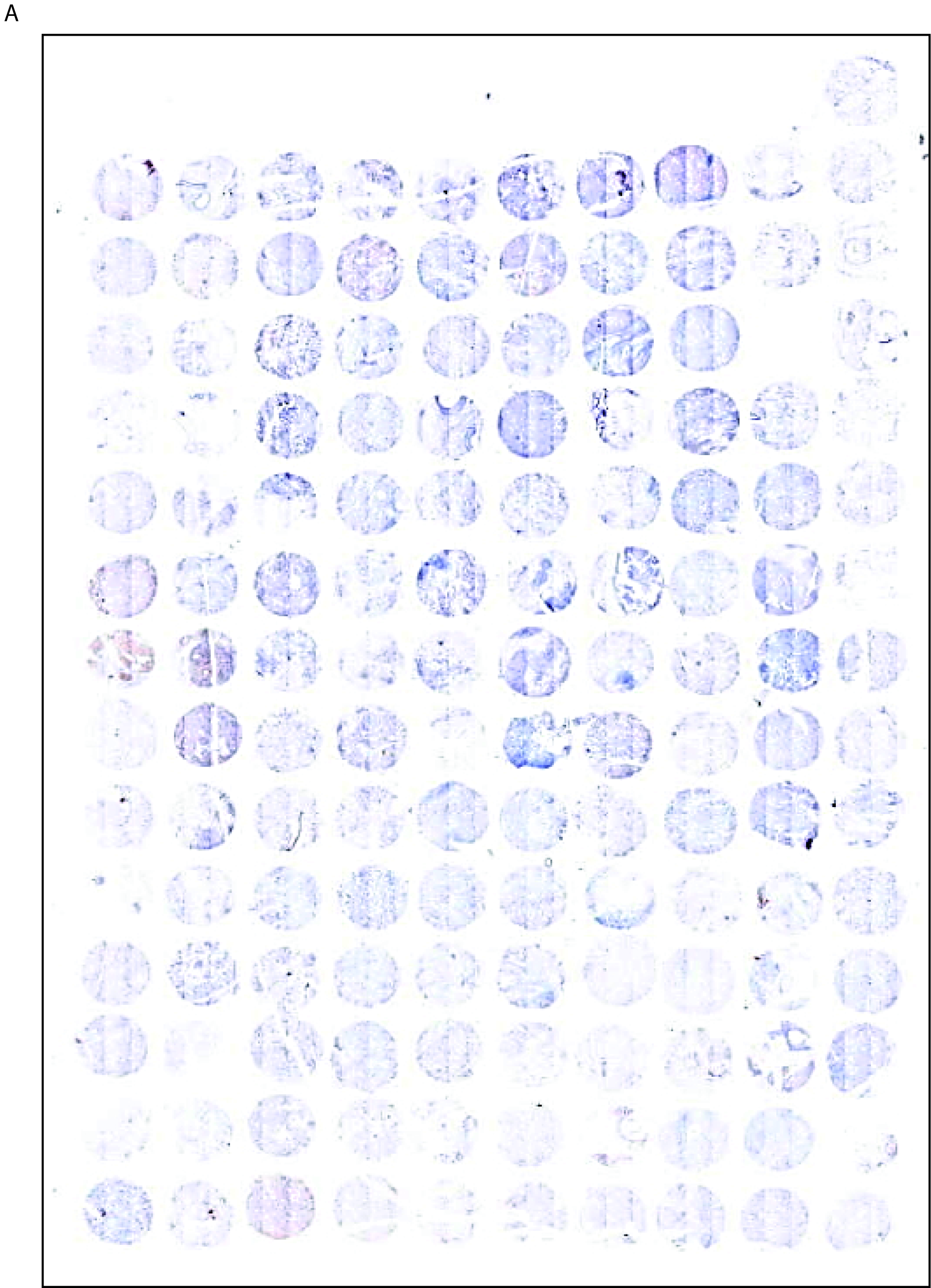

Supplement: Supplementary file 8 — Supplementary Figure 8 [file 41419_2023_5649_MOESM8_ESM.tif]
