# Supplementary material for: VPA mediates bidirectional regulation of cell cycle progression through the PPP2R2A-Chk1 signaling axis in response to HU
Source: Cell Death Dis. 2023 Feb 13;14(2):114. doi: 10.1038/s41419-023-05649-8 (PMC9925808; doi:10.1038/s41419-023-05649-8)
Supplement: Supplementary file 9 — Supplementary Figure legend [file 41419_2023_5649_MOESM9_ESM.docx]

**Supplementary Fig. S1 The bidirectional effects of VPA on cell cycle progression depend on Chk1 activation under HU treatment in cancer and normal cells.** (**A**) Survival fraction of MCF-10A cells after exposure to 0.5mM VPA and/or 2mM hydroxyurea (HU) was detected by clonogenic assay. Quantification was from three independent experiments. ***P* < 0.01. (**B**) MCF-10A cells were treated as stated in **A**. Cell cycle analysis of the DNA content of MCF-10A cells was analyzed by flow cytometry. Cell cycle analysis is shown as mean ± SD (n=3). Statistical significance is displayed for S cells. ***P* < 0.01. (**C**) MCF-7 cells were treated as depicted in **A**. After fixation, cells were counterstained with a pCDK1-Y-15 specific antibody (green). DAPI was used to visualize nuclei. Representative images are shown (scale bar, 10μm). Quantification of positive signal of pCDK1-Y-15 was from three independent experiments. ***P* < 0.01. (**D**) MCF-10A cells were treated as stated in **A**. Whole cell lysates were subjected to Western blotting and probed with pChk1-S317, pChk1-S345, Chk1, WEE1 and pCDK1-Y-15 antibodies. GAPDH was used as a loading control. (**E**) and (**F**) MCF-7 and 16HBE cells were subjected to cell cycle analysis in the presence of 0.5mM VPA, 2mM HU or/and 1.5µM Chk1 inhibitor (Chk1i) LY2603618. Cell cycle analysis of the DNA content of MCF-7 and 16HBE cells was analyzed by flow cytometry.

**Supplementary Fig. S2 The effects of VPA on the replication fork stability under HU treatment are Chk1 dependent.** (**A**) and (**B**) Representative images of γH2AX and pRPA2 (S4/8) foci in MCF-7 and 16HBE cells with or without VPA/HU in the presence or absence of Chk1 inhibition. Quantification was from three independent experiments. ***P* < 0.01. ns, not significant.

**Supplementary Fig. S3 Validation of working efficiency of LB-100.** (**A**) MCF-7 and 16HBE cells were treated with 2.5µM LB-100 for 48h, Whole cell lysates were subjected to Western blotting and probed with pTBK1 and TBK1 antibodies. GAPDH was used as a loading control.

**Supplementary Fig. S4 PPP2R2A regulates cancer cells survival.** (**A**) PPP2R2A deficient MCF-7 cells induced by two independent gRNAs were treated with VPA, HU, or the combination of VPA and HU, and was then subjected to clonogenic assay. Quantification was from three independent experiments. ns, not significant. **P* < 0.05, ***P* < 0.01. (**B**) MCF-7 cells were transduced with non-targeting gRNA (CTR gRNA) or PPP2R2A-targeting gRNAs (PPP2R2A gRNA#1 and PPP2R2A gRNA#2) and PPP2R2A deficiency was validated by Western blotting analysis.

**Supplementary Fig. S5 Expression of PPP2R2A and pChk1-S317 in rat breast tumor and spleen tissues.** (**A**) and (**B**) The rats with breast tumor were induced by DMBA. The expression of PPP2R2A and pChk1-S317 by immunohistochemistry staining in tumor and spleen tissues were shown. Notes: IOD of indicated proteins in immunohistochemistry photos was quantified. Each data point in the graph was from three independent experiments, ***P* < 0.01.

**Supplementary Fig. S6 PPP2R2A deficiency stabilizes Chk1.** (**A**) PPP2R2A deficiency leads to increase in Chk1 protein level, as detected by Western blotting analysis. MCF-7 cells transfected with two independent PPP2R2A-targeting siRNAs were treated with cycloheximide (50μg/ml) and cells were collected for Western blotting analysis at the indicated time points. Chk1 protein expression was quantified in three independent experiments. ***P* < 0.01.

**Supplementary Fig. S7 Expression of PPP2R2A in 140 cancer tissue microarray samples.** (**A**) The protein expression of PPP2R2A by immunohistochemistry staining in tumor tissues was shown. The bottom left corner is A1, the top right corner is J14, in order.

**Supplementary Fig. S8 Expression of pChk1-S317 in 140 cancer tissue microarray samples.** (**A**) The protein expression of pChk1-S317 by immunohistochemistry staining in tumor tissues was shown. The bottom left corner is A1, the top right corner is J14, in order.
