# Supplementary material for: VPA mediates bidirectional regulation of cell cycle progression through the PPP2R2A-Chk1 signaling axis in response to HU
Source: Cell Death Dis. 2023 Feb 13;14(2):114. doi: 10.1038/s41419-023-05649-8 (PMC9925808; doi:10.1038/s41419-023-05649-8)
Supplement: Supplementary file 10 — Supplementary Table S1 [file 41419_2023_5649_MOESM10_ESM.docx]

**Supp Table 1. Plasmids used in this study**

| Plasmids | Source | Identifier |
| --- | --- | --- |
| 3xflag-Homo-PPP2R2A | IGEbio | Plasmid #IG211912 |
| HA-Chk1 | Gift from Xingzhi, Xu | N/A |
| psPAX2 | Addgene | Plasmid #12260 |
| pMD2.G | Addgene | Plasmid #12259 |
| LentiCRISPR v2 | Addgene | Plasmid #52961 |
| Myc-PPP2R2A WT | Gift from [Xavier Graña](https://pubmed.ncbi.nlm.nih.gov/?term=Gra%C3%B1a+X&cauthor_id=34661528) | N/A |
| Myc-PPP2R2A K48E | Gift from [Xavier Graña](https://pubmed.ncbi.nlm.nih.gov/?term=Gra%C3%B1a+X&cauthor_id=34661528) | N/A |
| Myc-PPP2R2A L225A | Gift from [Xavier Graña](https://pubmed.ncbi.nlm.nih.gov/?term=Gra%C3%B1a+X&cauthor_id=34661528) | N/A |
| Myc-PPP2R2A H179A | Gift from [Xavier Graña](https://pubmed.ncbi.nlm.nih.gov/?term=Gra%C3%B1a+X&cauthor_id=34661528) | N/A |
| Myc-PPP2R2A N181A | Gift from [Xavier Graña](https://pubmed.ncbi.nlm.nih.gov/?term=Gra%C3%B1a+X&cauthor_id=34661528) | N/A |
| Myc-PPP2R2A D197K | Gift from [Xavier Graña](https://pubmed.ncbi.nlm.nih.gov/?term=Gra%C3%B1a+X&cauthor_id=34661528) | N/A |
| Myc-PPP2R2A V228A | Gift from [Xavier Graña](https://pubmed.ncbi.nlm.nih.gov/?term=Gra%C3%B1a+X&cauthor_id=34661528) | N/A |
| Myc-PPP2R2A S247A | Gift from [Xavier Graña](https://pubmed.ncbi.nlm.nih.gov/?term=Gra%C3%B1a+X&cauthor_id=34661528) | N/A |
| Myc-PPP2R2A F280A | Gift from [Xavier Graña](https://pubmed.ncbi.nlm.nih.gov/?term=Gra%C3%B1a+X&cauthor_id=34661528) | N/A |
| Myc-PPP2R2A E283A | Gift from [Xavier Graña](https://pubmed.ncbi.nlm.nih.gov/?term=Gra%C3%B1a+X&cauthor_id=34661528) | N/A |
