# Supplementary material for: VPA mediates bidirectional regulation of cell cycle progression through the PPP2R2A-Chk1 signaling axis in response to HU
Source: Cell Death Dis. 2023 Feb 13;14(2):114. doi: 10.1038/s41419-023-05649-8 (PMC9925808; doi:10.1038/s41419-023-05649-8)
Supplement: Supplementary file 11 — Supplementary Table S2 [file 41419_2023_5649_MOESM11_ESM.docx]

**Supp Table 2. Sequences of siRNAs used in this study**

| **siRNA** | **Sequence** |
| --- | --- |
| siControl | UUCUCCGAACGUGUCACGUTT |
| siPPP2R2A-1 | GGAUGGAAGGUAUAGAGAUTT |
| siPPP2R2A-2 | GCCAAUAUGGAAGAGCUAATT |
| siHDAC1 | CAGCGACUGUUUGAGAACC |
| siHDAC2 | GCGGAUAGCUUGUGAUGAA |
