# Supplementary material for: VPA mediates bidirectional regulation of cell cycle progression through the PPP2R2A-Chk1 signaling axis in response to HU
Source: Cell Death Dis. 2023 Feb 13;14(2):114. doi: 10.1038/s41419-023-05649-8 (PMC9925808; doi:10.1038/s41419-023-05649-8)
Supplement: Supplementary file 12 — Supplementary Table S3 [file 41419_2023_5649_MOESM12_ESM.docx]

**Supp Table 3. Sequences of gRNAs used in this study**

| **gRNA** | **Sequence** |
| --- | --- |
| Non-targeting control gRNA | GTATTACTGATATTGGTGGG |
| PPP2R2A#1 | GCCTCAACCATTAGATCCAT |
| PPP2R2A#2 | GCCTATGGATCTAATGGTTG |
