# Supplementary material for: VPA mediates bidirectional regulation of cell cycle progression through the PPP2R2A-Chk1 signaling axis in response to HU
Source: Cell Death Dis. 2023 Feb 13;14(2):114. doi: 10.1038/s41419-023-05649-8 (PMC9925808; doi:10.1038/s41419-023-05649-8)
Supplement: Supplementary file 13 — Supplementary Table S4 [file 41419_2023_5649_MOESM13_ESM.docx]

**Supp Table 4. Sequences of primers used in this study**

| **Primers** | **Sequences** |
| --- | --- |
| **GAPDH** | Forward GAGAAGGCTGGGGCTCATTT |
|  | Reverse AGTGATGGCATGGACTGTGG |
| **PPP2R2A** | Forward CCACCTTTATCTCCTGTTGC |
|  | Reverse TTTCTCAGGTGAAAGGAGCAG |
