# Supplementary material for: VPA mediates bidirectional regulation of cell cycle progression through the PPP2R2A-Chk1 signaling axis in response to HU
Source: Cell Death Dis. 2023 Feb 13;14(2):114. doi: 10.1038/s41419-023-05649-8 (PMC9925808; doi:10.1038/s41419-023-05649-8)
Supplement: Supplementary file 17 — Original western blots [file 41419_2023_5649_MOESM17_ESM.pdf]

Figure 1G

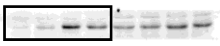

MCF-7 pChk1-S317

Figure 1G

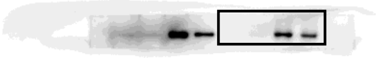

MCF-7 pChk1-S345

Figure 1G

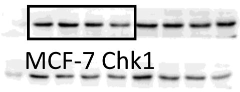

MCF-7 Chk1

Figure 1G

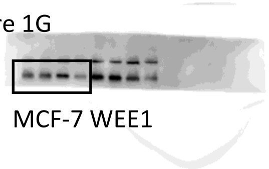

MCF-7 WEE1

Figure 1G

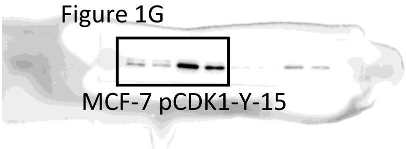

MCF-7 pCDK1-Y-15

Figure 1G

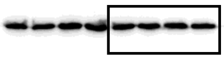

MCF-7 GAPDH

Figure 1I

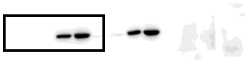

16HBE pChk1-S317

Figure 1I

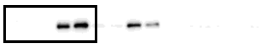

16HBE pChk1-S345

Figure 1I

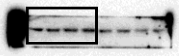

16HBE Chk1

Figure 1I

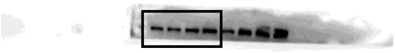

16HBE WEE1

Figure 1I

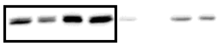

16HBE pCDK1-Y-15

Figure 1I

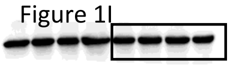

16HBE GAPDH

Figure 1O

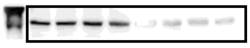

MCF-7+Chk1i Chk1

Figure 1O

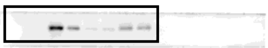

MCF-7+Chk1i pChk1-S317

Figure 1O

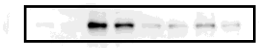

MCF-7+Chk1i pChk1-S345

Figure 1O

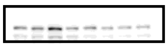

MCF-7+Chk1i WEE1

Figure 1O

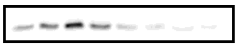

MCF-7+Chk1i pCDK1-Y-15

Figure 1O

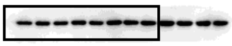

MCF-7+Chk1i GAPDH

Figure 1O

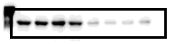

16HBE+Chk1i Chk1

Figure 1O

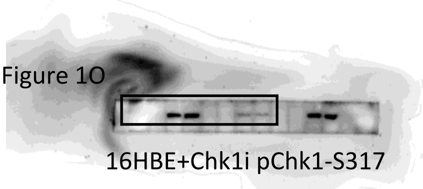

16HBE+Chk1i pChk1-S317

Figure 1O

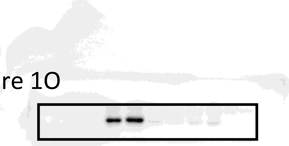

16HBE+Chk1i pChk1-S345

Figure 1O

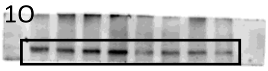

16HBE+Chk1i WEE1

Figure 1O

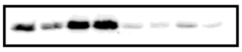

16HBE+Chk1i pCDK1-Y-15

Figure 1O

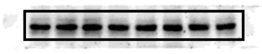

16HBE+Chk1i GAPDH

Figure 2E

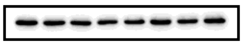

MCF-7+PP2Ai PP2A

Figure 2E

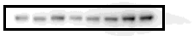

MCF-7+PP2Ai Chk1

Figure 2E

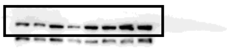

MCF-7+PP2Ai WEE1

Figure 2E

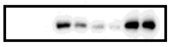

MCF-7+PP2Ai pChk1-S345

Figure 2E

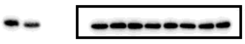

MCF-7+PP2Ai GAPDH

Figure 2E

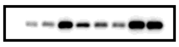

MCF-7+PP2Ai pCDK1-Y-15

Figure 2E

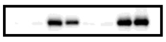

MCF-7+PP2Ai pChk1-S317

Figure 2F

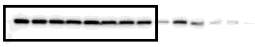

16HBE+PP2Ai PP2A

Figure 2F

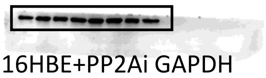

16HBE+PP2Ai GAPDH

Figure 2F

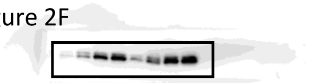

16HBE+PP2Ai pCDK1-Y-15

Figure 2F

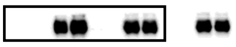

16HBE+PP2Ai pChk1-S345

Figure 2F

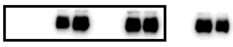

16HBE+PP2Ai pChk1-S317

Figure 2F

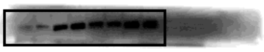

16HBE+PP2Ai WEE1

Figure 2F

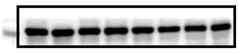

16HBE+PP2Ai Chk1

Figure 3A

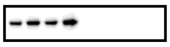

MCF-7+siPPP2R2A-1 PPP2R2A

Figure 3A

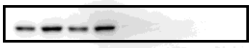

MCF-7+siPPP2R2A-2 PPP2R2A

Figure 3A

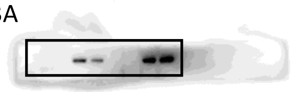

MCF-7+siPPP2R2A-1 pChk1-S317

Figure 3A

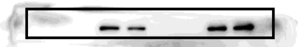

MCF-7+siPPP2R2A-1 pChk1-S345

Figure 3A

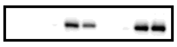

MCF-7+siPPP2R2A-2 pChk1-S317

Figure 3A

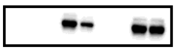

MCF-7+siPPP2R2A-2 pChk1-S345

Figure 3A

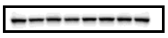

MCF-7+siPPP2R2A-2 Chk1

Figure 3A

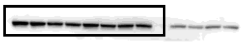

MCF-7+siPPP2R2A-1 Chk1

Figure 3A

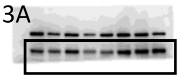

MCF-7+siPPP2R2A-1 WEE1

Figure 3A

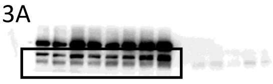

MCF-7+siPPP2R2A-2 WEE1

Figure 3A

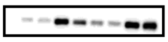

MCF-7+siPPP2R2A-1 pCDK1-Y-15

Figure 3A

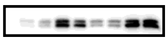

MCF-7+siPPP2R2A-2 pCDK1-Y-15

Figure 3A

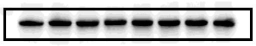

MCF-7+siPPP2R2A-1 GAPDH

Figure 3A

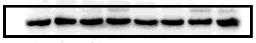

MCF-7+siPPP2R2A-2 GAPDH

Figure 3E

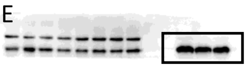

MCF-7+PPP2R2A KO GAPDH

Figure 3E

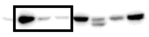

MCF-7+PPP2R2A KO

Figure 3B

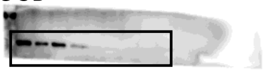

16HBE+siPPP2R2A-1 PPP2R2A

Figure 3B

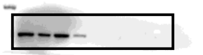

16HBE+siPPP2R2A-2 PPP2R2A

Figure 3B

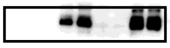

16HBE+siPPP2R2A-1 pChk1-S317

Figure 3B

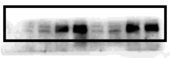

16HBE+siPPP2R2A-1 pChk1-S345

Figure 3B

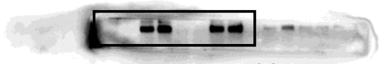

16HBE+siPPP2R2A-2 pChk1-S317

Figure 3B

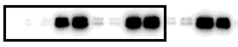

16HBE+siPPP2R2A-2 pChk1-S345

Figure 3B

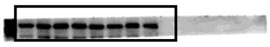

16HBE+siPPP2R2A-1 Chk1

Figure 3B

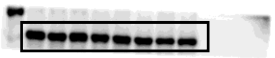

16HBE+siPPP2R2A-2 Chk1

Figure 3B

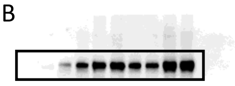

16HBE+siPPP2R2A-1 WEE1

Figure 3B

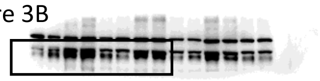

16HBE+siPPP2R2A-2 WEE1

Figure 3B

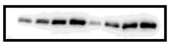

16HBE+siPPP2R2A-1 pCDK1-Y-15

Figure 3B

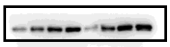

16HBE+siPPP2R2A-2 pCDK1-Y-15

Figure 3B

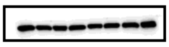

16HBE+siPPP2R2A-2 GAPDH

Figure 3B

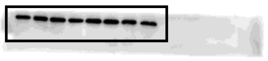

16HBE+siPPP2R2A-1 GAPDH

Figure 3E

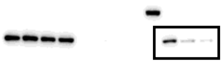

16HBE+siPPP2R2A

Figure 3E

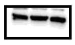

16HBE+siPPP2R2A GAPDH

Figure 5A

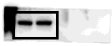

Input-Chk1

Figure 5A

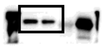

Input-Chk1

Figure 5A

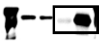

IP-Chk1

Figure 5A

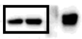

Input-PPP2R2A

Figure 5A

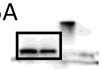

Input-PPP2R2A

Figure 5A

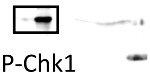

IP-Chk1

Figure 5A

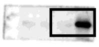

IP-PPP2R2A

Figure 5A

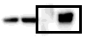

IP-PPP2R2A

Figure 5B

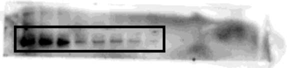

CHX-PPP2R2A KO2-PPP2R2A

Figure 5B

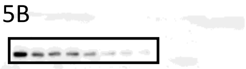

CHX-PPP2R2A KO1-PPP2R2A

Figure 5B

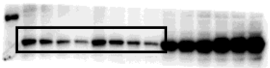

CHX-PPP2R2A KO2-Chk1

Figure 5B

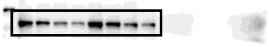

CHX-PPP2R2A KO1-Chk1

Figure 5B

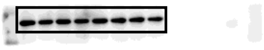

CHX-PPP2R2A KO1 GAPDH

Figure 5B

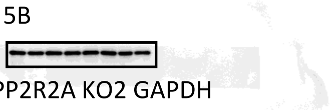

CHX-PPP2R2A KO2 GAPDH

Figure 5C

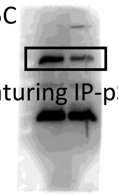

Denaturing IP-pS/TP

Figure 5C

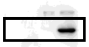

Denaturing IP Input-Flag

Figure 5C

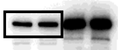

Denaturing IP Input-HA

Figure 5C

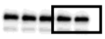

Denaturing IP IP-HA

Figure 5C

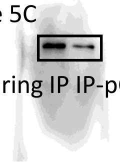

Denaturing IP IP-pChk1-S317

Figure 5C

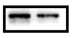

Denaturing IP IP-pChk1-S345

Figure 5D

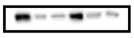

PPP2R2A KO HU PPP2R2A

Figure 5D

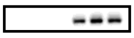

PPP2R2A KO HU pChk1-S317

Figure 5D

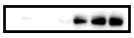

PPP2R2A KO HU pChk1-S345

Figure 5D

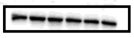

PPP2R2A KO HU Chk1

Figure 5D

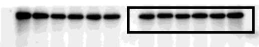

PPP2R2A KO HU GAPDH

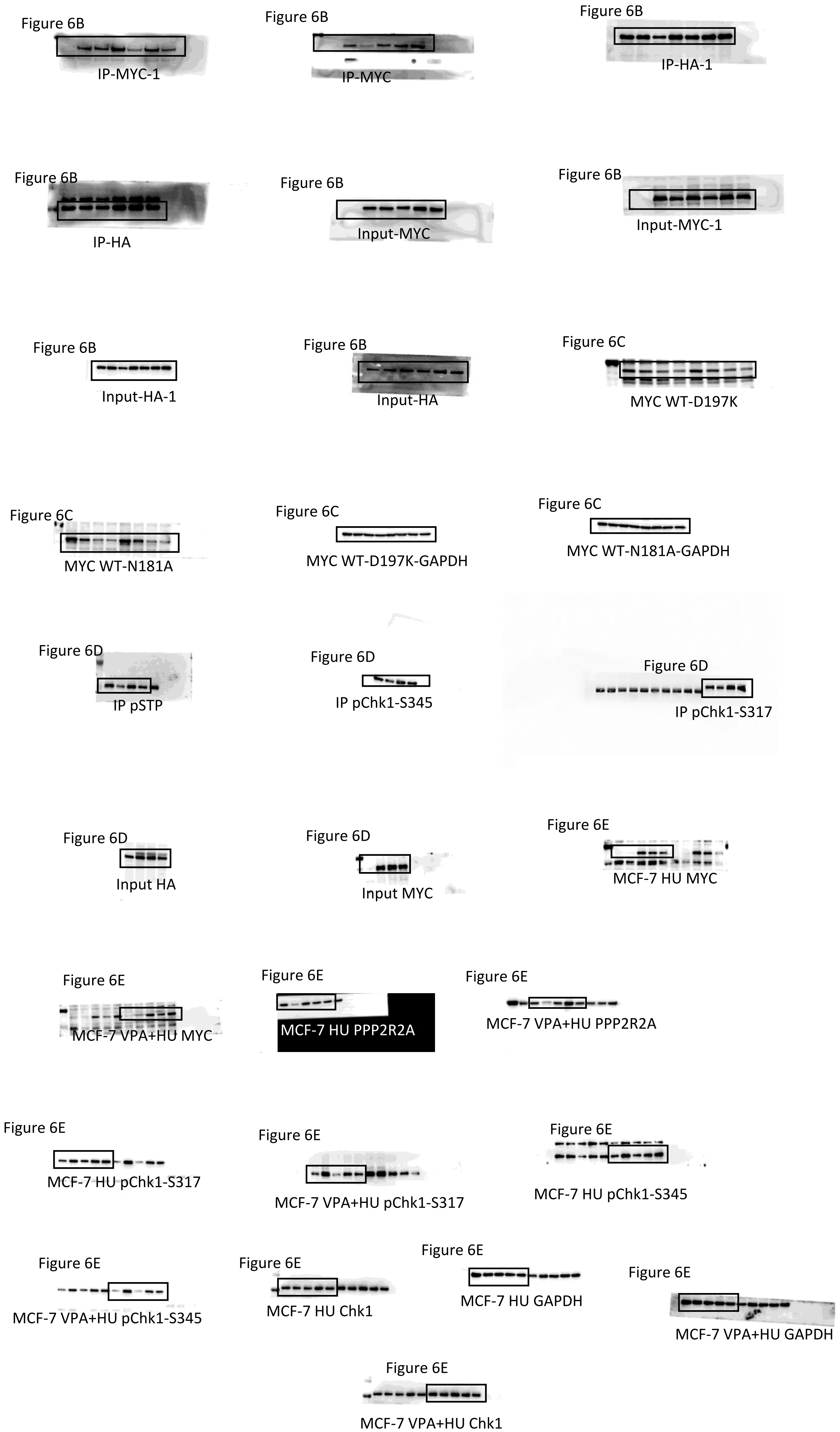

Figure 6B
IP-MYC-1
Figure 6B
IP-MYC
Figure 6B
IP-HA-1
Figure 6B
IP-HA
Figure 6B
Input-MYC
Figure 6B
Input-MYC-1
Figure 6B
Input-HA-1
Figure 6B
Input-HA
Figure 6C
MYC WT-D197K
Figure 6C
MYC WT-N181A
Figure 6C
MYC WT-D197K-GAPDH
Figure 6C
MYC WT-N181A-GAPDH
Figure 6D
IP pSTP
Figure 6D
IP pChk1-S345
Figure 6D
IP pChk1-S317
Figure 6D
Input HA
Figure 6D
Input MYC
Figure 6E
MCF-7 HU MYC
Figure 6E
MCF-7 VPA+HU MYC
Figure 6E
MCF-7 HU PPP2R2A
Figure 6E
MCF-7 VPA+HU PPP2R2A
Figure 6E
MCF-7 HU pChk1-S317
Figure 6E
MCF-7 VPA+HU pChk1-S317
Figure 6E
MCF-7 HU pChk1-S345
Figure 6E
MCF-7 VPA+HU pChk1-S345
Figure 6E
MCF-7 HU Chk1
Figure 6E
MCF-7 HU GAPDH
Figure 6E
MCF-7 VPA+HU GAPDH
Figure 6E
MCF-7 VPA+HU Chk1

Figure 6E

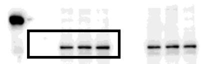

16HBE HU MYC

Figure 6E

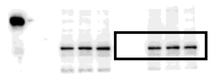

16HBE VPA+HU MYC

Figure 6E

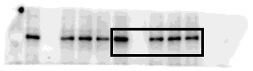

16HBE HU PPP2R2A

Figure 6E

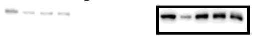

16HBE VPA+HU PPP2R2A

Figure 6E

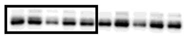

16HBE VPA+HU pChk1-S345

Figure 6E

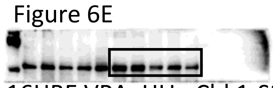

16HBE VPA+HU pChk1-S317

Figure 6E

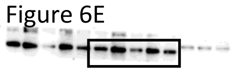

16HBE HU pChk1-S317

Figure 6E

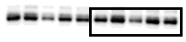

16HBE HU pChk1-S345

Figure 6E

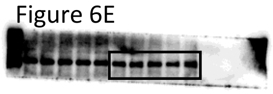

16HBE VPA+HU Chk1

Figure 6E

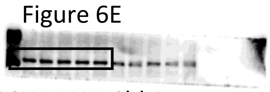

16HBE HU Chk1

Figure 6E

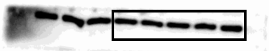

16HBE VPA+HU GAPDH

Figure 6E

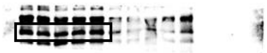

16HBE HU GAPDH

Figure 7E

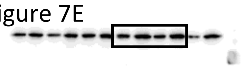

MCF-7 PPP2R2A

Figure 7E

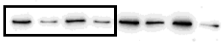

MCF-7 HDAC1

Figure 7E

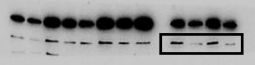

MCF-7 HDAC2

Figure 7E

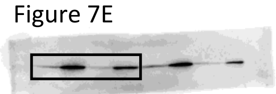

MCF-7 Acetyl-H3

Figure 7E

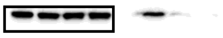

MCF-7 GAPDH

Figure 7G

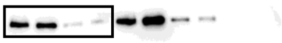

MCF-7 HDAC1

Figure 7G

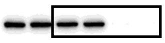

MCF-7 HDAC2

Figure 7G

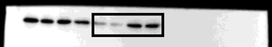

MCF-7 Acetyl-H3

Figure 7G

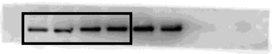

MCF-7 PPP2R2A

Figure 7G

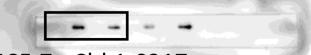

MCF-7 pChk1-S317

Figure 7G

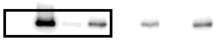

MCF-7 pChk1-S345

Figure 7G

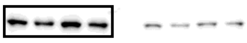

MCF-7 Chk1

Figure 7G

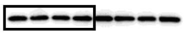

MCF-7 GAPDH

Figure 7F

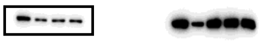

16HBE PPP2R2A

Figure 7F

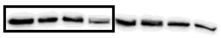

16HBE HDAC1

Figure 7F

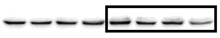

16HBE HDAC2

Figure 7F

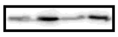

16HBE Acetyl-H3

Figure 7F

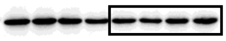

16HBE GAPDH

Figure 7H

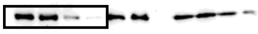

16HBE HDAC1

Figure 7H

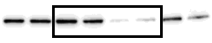

16HBE HDAC2

Figure 7H

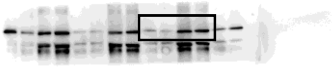

16HBE Acetyl-H3

Figure 7H

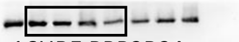

16HBE PPP2R2A

Figure 7H

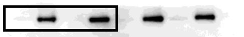

16HBE Chk1-S317

Figure 7H

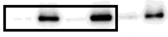

16HBE Chk1-S345

Figure 7H

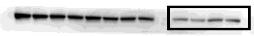

16HBE Chk1

Figure 7H

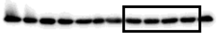

16HBE GAPDH

Supplementary Figure 1D

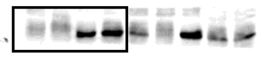

MCF-10A pChk1-S317

Supplementary Figure 1D

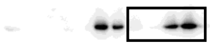

MCF-10A pChk1-S345

Supplementary Figure 1D

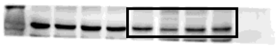

MCF-10A Chk1

Supplementary Figure 1D

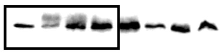

MCF-10A WEE1

Supplementary Figure 1D

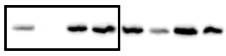

MCF-10A pCDK1-Y-15

mentary Figure 1D

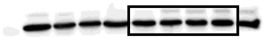

MCF-10A GAPDH

Supplementary Figure 3A

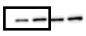

16HBE PP2Ai pTBK1

Supplementary Figure 3A

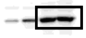

16HBE PP2Ai GAPDH

Supplementary Figure 3A

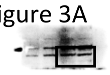

16HBE PP2Ai TBK1

Supplementary Figure 3A

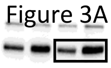

MCF-7 PP2Ai pTBK1

Supplementary Figure 3A

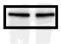

MCF-7 PP2Ai TBK1

Supplementary Figure 3A

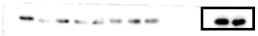

MCF-7 PP2Ai GAPDH

Supplementary Figure 4A

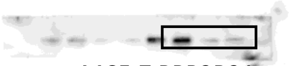

MCF-7 PPP2R2A

Supplementary Figure 4A

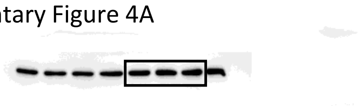

MCF-7 GAPDH

Supplementary Figure 6A

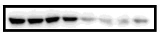

MCF-7 siPPP2R2A-1 PPP2R2A

Supplementary Figure 6A

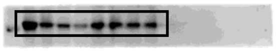

MCF-7 siPPP2R2A-1 Chk1

Supplementary Figure 6A

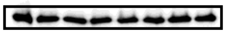

MCF-7 siPPP2R2A-1 GAPDH

Supplementary Figure 6A

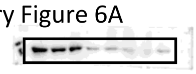

MCF-7 siPPP2R2A-2 PPP2R2A

Supplementary Figure 6A

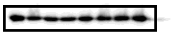

MCF-7 siPPP2R2A-2 GAPDH

Supplementary Figure 6A

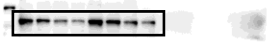

MCF-7 siPPP2R2A-2 Chk1
