# Supplementary material for: VPA mediates bidirectional regulation of cell cycle progression through the PPP2R2A-Chk1 signaling axis in response to HU
Source: Cell Death Dis. 2023 Feb 13;14(2):114. doi: 10.1038/s41419-023-05649-8 (PMC9925808; doi:10.1038/s41419-023-05649-8)
Supplement: Supplementary file 18 — Author Contribution Statement [file 41419_2023_5649_MOESM18_ESM.docx]

Benyu Su performed the majority of the laboratory work, the analysis of the data and the writing of the manuscript; Chenyang Qi, Zhongwei Zhang, Junxiao Wang, Fengmei Zhang, David Lim and Chao Dong provided analysis of data, statistical analysis; David Lim provided language editing; Chao Dong and Zhihui Feng were involved in manuscript writing, financial support. Zhihui Feng was involved in conception and design. All authors read and approved the final paper.
