# Supplementary material for: VPA mediates bidirectional regulation of cell cycle progression through the PPP2R2A-Chk1 signaling axis in response to HU
Source: Cell Death Dis. 2023 Feb 13;14(2):114. doi: 10.1038/s41419-023-05649-8 (PMC9925808; doi:10.1038/s41419-023-05649-8)
Supplement: Supplementary file 19 — Agreement from all authors [file 41419_2023_5649_MOESM19_ESM.pdf]

主　题:	回复：agree	
发件人:	"飞得更高" <1577963640@qq.com>	2023-2-2 12:24:48
收件人:	"凤志慧发送垃圾邮件" <fengzhihui@sdu.edu.cn>	

agree

------------------ 原始邮件 ------------------

发件人: "凤志慧发送垃圾邮件" <fengzhihui@sdu.edu.cn>;
发送时间: 2023年2月2日(星期四) 中午12:03
收件人: "飞得更高"<1577963640@qq.com>;"david lim"<d.lim3@westernsydney.edu.au>;"齐晨阳"<1445673950@qq.com>;"zhongwei8721"<zhongwei8721@163.com>;"2870031633"<2870031633@qq.com>;"fengmeizhang2003"<fengmeizhang2003@sdu.edu.cn>;"chaodong"<chaodong@sdu.edu.cn>;
主题: agree

Dear co-authors,
In this article "VPA mediates bidirectional regulation of cell cycle progression through the PPP2R2A-Chk1 signaling axis in response to HU", due to my mistake, I did not add Dr. David Lim into the initial submission system as co-author. Now he was added as a co-author in current manuscript based on his contributions in manuscript writing, language editing and data analysis. If you agree, please reply "agree".
Many thanks.
Yours sincerely,

Zhihui Feng

主　题:	Re: [External] agree
发件人:	"David Lim" <David.Lim@westernsydney.edu.au> 2023-2-2 12:04:07
收件人:	"凤志慧发送垃圾邮件" <fengzhihui@sdu.edu.cn>, "飞得更高" <1577963640@qq.com>, "齐晨阳" <1445673950@qq.com>, "zhongwei8721@163.com" <zhongwei8721@163.com>, "2870031633@qq.com" <2870031633@qq.com>, "fengmeizhang2003@sdu.edu.cn" <fengmeizhang2003@sdu.edu.cn>, "chaodong@sdu.edu.cn" <chaodong@sdu.edu.cn>

agreed

From: 凤志慧发送垃圾邮件 <fengzhihui@sdu.edu.cn>
Sent: Thursday, 2 February 2023 3:03 PM
To: 飞得更高 <1577963640@qq.com>; David Lim <David.Lim@westernsydney.edu.au>; 齐晨阳 <1445673950@qq.com>; zhongwei8721@163.com <zhongwei8721@163.com>; 2870031633@qq.com <2870031633@qq.com>; fengmeizhang2003@sdu.edu.cn <fengmeizhang2003@sdu.edu.cn>; chaodong@sdu.edu.cn <chaodong@sdu.edu.cn>
Subject: [External] agree

Dear co-authors,
In this article "VPA mediates bidirectional regulation of cell cycle progression through the PPP2R2A-Chk1 signaling axis in response to HU", due to my mistake, I did not add Dr. David Lim into the initial submission system as co-author. Now he was added as a co-author in current manuscript based on his contributions in manuscript writing, language editing and data analysis. If you agree, please reply "agree".
Many thanks.
Yours sincerely,
Zhihui Feng

主　题:	agree	
发件人:	"齐晨阳" <1445673950@qq.com>	2023-2-2 12:31:11
收件人:	"凤志慧发送垃圾邮件" <fengzhihui@sdu.edu.cn>	

agree

---原始邮件---

发件人：凤志慧发送垃圾邮件"

发送时间："undefined"

收件人："飞得更高"<1577963640@qq.com>,"david lim","齐晨阳"<1445673950@qq.com>,"zhongwei8721","2870031633"<2870031633@qq.com>,"fengmeizhang2003","chaodong"

主题：agree

Dear co-authors,
In this article "VPA mediates bidirectional regulation of cell cycle progression through the PPP2R2A-Chk1 signaling axis in response to HU”, due to my mistake, I did not add Dr. David Lim into the initial submission system as co-author. Now he was added as a co-author in current manuscript based on his contributions in manuscript writing, language editing and data analysis. If you agree, please reply "agree".
Many thanks.
Yours sincerely,

Zhihui Feng

主　题:	回复：agree
发件人:	"张中伟" <zhongwei8721@163.com> 2023-2-2 12:08:13
收件人:	fengzhihui <fengzhihui@sdu.edu.cn>
抄　送:	1577963640 <1577963640@qq.com>, "d.lim3" <d.lim3@westernsydney.edu.au>, 1445673950 <1445673950@qq.com>, 2870031633 <2870031633@qq.com>, fengmeizhang2003 <fengmeizhang2003@sdu.edu.cn>, chaodong <chaodong@sdu.edu.cn>

agree.

zhongwei8721@163.com

邮箱：zhongwei8721@163.com

---- 回复的原邮件 ----

发件人	凤志慧发送垃圾邮件<fengzhihui@sdu.edu.cn>
日期	2023年02月02日 12:03
收件人	飞得更高<1577963640@qq.com>、david lim<d.lim3@westernsydney.edu.au>、齐晨阳<1445673950@qq.com>、zhongwei8721@163.com<zhongwei8721@163.com>、2870031633@qq.com<2870031633@qq.com>、fengmeizhang2003@sdu.edu.cn<fengmeizhang2003@sdu.edu.cn>、chaodong@sdu.edu.cn<chaodong@sdu.edu.cn>
主题	agree

Dear co-authors,
In this article "VPA mediates bidirectional regulation of cell cycle progression through the PPP2R2A-Chk1 signaling axis in response to HU", due to my mistake, I did not add Dr. David Lim into the initial submission system as co-author. Now he was added as a co-author in current manuscript based on his contributions in manuscript writing, language editing and data analysis. If you agree, please reply "agree".
Many thanks.
Yours sincerely,

Zhihui Feng

主　题:	回复：agree	
发件人:	"王峻潇" <2870031633@qq.com>	2023-2-2 12:04:07
收件人:	"凤志慧发送垃圾邮件" <fengzhihui@sdu.edu.cn>	

agree

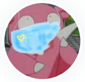

王峻潇

2870031633@qq.com

------------------ 原始邮件 ------------------

发件人: "凤志慧发送垃圾邮件" <fengzhihui@sdu.edu.cn>;
发送时间: 2023年2月2日(星期四) 中午12:03
收件人: "飞得更高"<1577963640@qq.com>;"david lim"<d.lim3@westernsydney.edu.au>;"齐晨阳"<1445673950@qq.com>;"zhongwei8721"<zhongwei8721@163.com>;"王峻潇" <2870031633@qq.com>;"fengmeizhang2003"<fengmeizhang2003@sdu.edu.cn>;"chaodong"<chaodong@sdu.edu.cn>;
主题: agree

Dear co-authors,
In this article "VPA mediates bidirectional regulation of cell cycle progression through the PPP2R2A-Chk1 signaling axis in response to HU”, due to my mistake, I did not add Dr. David Lim into the initial submission system as co-author. Now he was added as a co-author in current manuscript based on his contributions in manuscript writing, language editing and data analysis. If you agree, please reply "agree".
Many thanks.
Yours sincerely,

Zhihui Feng

主　题:	回复：agree
发件人:	"fengmeizhang2003@sdu.edu.cn" <fengmeizhang2003@sdu.edu.cn>　　2023-2-2 12:30:09
收件人:	"凤志慧发送垃圾邮件" <fengzhihui@sdu.edu.cn>

agree

发自我的手机

-------- 原始邮件 --------
发件人： 凤志慧发送垃圾邮件 <fengzhihui@sdu.edu.cn>
日期： 2023年2月2日周四 中午12:03
收件人： 飞得更高 <1577963640@qq.com>, david lim <d.lim3@westernsydney.edu.au>, 齐晨阳 <1445673950@qq.com>, zhongwei8721@163.com, 2870031633@qq.com, fengmeizhang2003@sdu.edu.cn, chaodong@sdu.edu.cn
主 题： agree

Dear co-authors,

In this article "VPA mediates bidirectional regulation of cell cycle progression through the PPP2R2A-Chk1 signaling axis in response to HU", due to my mistake, I did not add Dr. David Lim into the initial submission system as co-author. Now he was added as a co-author in current manuscript based on his contributions in manuscript writing, language editing and data analysis. If you agree, please reply "agree". Many thanks.

Yours sincerely,

Zhihui Feng

主　题:	Re:agree	
发件人:	"Chao Dong" <chaodong@sdu.edu.cn>	2023-2-2 12:08:05
收件人:	"凤志慧发送垃圾邮件" <fengzhihui@sdu.edu.cn>	

agree.

Chao Dong

School of Public Health, Shandong Uni.

chaodong@sdu.edu.cn

---- Replied Message ----

From	凤志慧发送垃圾邮件<fengzhihui@sdu.edu.cn>
Date	02/2/2023 11:00
To	飞得更高<1577963640@qq.com> ,<br>david lim<d.lim3@westernsydney.edu.au> ,<br>齐晨阳<1445673950@qq.com> ,<br><zhongwei8721@163.com> ,<br><2870031633@qq.com> ,<br><fengmeizhang2003@sdu.edu.cn> ,<br><chaodong@sdu.edu.cn>
Subject	agree

Dear co-authors,

In this article "VPA mediates bidirectional regulation of cell cycle progression through the PPP2R2A-Chk1 signaling axis in response to HU", due to my mistake, I did not add Dr. David Lim into the initial submission system as co-author. Now he was added as a co-author in current manuscript based on his contributions in manuscript writing, language editing and data analysis. If you agree, please reply "agree". Many thanks.

Yours sincerely,

Zhihui Feng
