# Supplementary material for: VPA mediates bidirectional regulation of cell cycle progression through the PPP2R2A-Chk1 signaling axis in response to HU
Source: Cell Death Dis. 2023 Feb 13;14(2):114. doi: 10.1038/s41419-023-05649-8 (PMC9925808; doi:10.1038/s41419-023-05649-8)
Supplement: Supplementary file 20 — Change of authorship request form - Journals [file 41419_2023_5649_MOESM20_ESM.pdf]

SPRINGER NATURE

Change of authorship request form - Journals (pre-acceptance)

Important information. Please read.

- This form should be used by authors to request any change in authorship (adding/deleting authors) including changes in corresponding authors. This form should not be used for name changes. Please fully complete all sections. Use black ink and block capitals and provide each author's full name with the given name first followed by the family name.

- By signing this declaration, all authors guarantee that the order of the authors are in accordance with their scientific contribution, if applicable as different conventions apply per discipline, and that only authors have been added who made a meaningful contribution to the work.

- Please note, in author collaborations where there is formal agreement for representing the collaboration, it is sufficient for the representative or legal guarantor (usually the corresponding author) to complete and sign the Authorship Change Form on behalf of all authors, **next to the added/removed author(s)**. **(Complete Section 3, followed by Section 6.)**
  In author collaborations where there is no formal agreement for representing the collaboration and **there are more than 10 authors**, one may sign for all, provided the signer appends correspondence that attests that each of the authors have agreed to the change **and the added/removed authors sign the form**. **(Complete Section 3, followed by Section 6.)**

- Please note, we cannot investigate or mediate any authorship disputes. If you are unable to obtain agreement from all authors (including those who you wish to be removed) you must refer the matter to your institution(s) for investigation. Please inform us if you need to do this.

- If you are not able to return a fully completed form within **30 days** of the date that it was sent to the author requesting the change, we may have to withdraw your manuscript. We cannot publish manuscripts where authorship has not been agreed by all authors (including those who have been removed).

- Incomplete forms will be rejected.

- Please return/upload this form, fully completed, to the Journals Editorial Office. The Journal and/or Publisher will consider the information you have provided to decide whether to approve the proposed change in authorship. We may decide to contact your institution for more information or undertake a further investigation, if appropriate, before making a final decision.

SPRINGER NATURE

Change of authorship request form - Journals (pre-acceptance)

Section 1: Please provide the current title of manuscript

Manuscript ID no.: CDDIS-22-4258RR

Title: VPA mediates bidirectional regulation of cell cycle progression through the PPP2R2A-Chk1 signalling axis in response to HU

Section 2: Please provide the previous authorship, in the order shown on the manuscript before the changes were introduced. Please indicate the corresponding author by adding (CA) behind the name.

	First name(s)	Family name	ORCID or SCOPUS id, if available
1st author	Benyu	Su	
2nd author	Chenyang	Qi	
3rd author	Zhongwei	Zhang	
4th author	Junxiao	Wang	
5th author	Fengmei	Zhang	
6th author	Chao	Dong	
7th author	Zhihui	Feng	
8th author			
9th author			
10th author			

Please use an additional sheet if there are more than 10 authors.

SPRINGER NATURE Springer Nature is one of the world's leading global research, educational and professional publishers, created in May 2015 through the combination of Nature Publishing Group, Palgrave Macmillan, Macmillan Education and Springer Science+Business Media.

SPRINGER NATURE

Change of authorship request form - Journals (pre-acceptance)

Section 3: Please provide a justification for change. Please use this section to explain your reasons for changing the authorship of your manuscript, e.g. what necessitated the change in authorship? Please refer to the (journal) policy pages for more information about authorship. Please explain why omitted authors were not originally included and/or why authors were removed on the submitted manuscript.

In this article "VPA mediates bidirectional regulation of cell cycle progression through the PPP2R2A-Chk1 signaling axis in response to HU”, due to my mistake, I did not add Dr. David Lim into the initial submission system as co-author. Now he was added as a co-author in current manuscript based on his contributions in manuscript writing, language editing and data analysis.

Section 4: Proposed new authorship. Please provide your new authorship list in the order you would like it to appear on the manuscript. Please indicate the corresponding author by adding (CA) behind the name. If the Corresponding Author has changed, please indicate the reason under section 3.

	First name(s)	Family name (this name will appear in full on the final publication and will be searchable in various abstract and indexing databases)	Affiliated institute	E-mail address
1st author	Benyu	Su	Cheeloo College of Medicine, Shandong University	1577963640@qq.com
2nd author	David	Lim	School of Health Sciences, Western Sydney University	David.Lim@westernsydney.edu.au
3rd author	Chenyang	Qi	Cheeloo College of Medicine, Shandong University	1445673950@qq.com
4th author	Zhongwei	Zhang	Cheeloo College of Medicine, Shandong University	zhongwei8721@163.com
5th author	Junxiao	Wang	Cheeloo College of Medicine, Shandong University	2870031633@qq.com
6th author	Fengmei	Zhang	Cheeloo College of Medicine, Shandong University	fengmeizhang2003@sdu.edu.cn
7th author	Chao	Dong	Cheeloo College of Medicine, Shandong University	chaodong@sdu.edu.cn
8th author	Zhihui	Feng	Cheeloo College of Medicine, Shandong University	fengzhihui@sdu.edu.cn
9th author				
10th author				

Please use an additional sheet if there are more than 10 authors.

SPRINGER NATURE Springer Nature is one of the world’s leading global research, educational and professional publishers, created in May 2015 through the combination of Nature Publishing Group, Palgrave Macmillan, Macmillan Education and Springer Science+Business Media.

SPRINGER NATURE

Change of authorship request form - Journals (pre-acceptance)

Section 5: Author contribution, Acknowledgement and Disclosures. Please use this section to provide a new disclosure statement and, if appropriate, acknowledge any contributors who have been removed as authors and ensure you state what contribution any new authors made (if applicable per the journal or book (series) policy). Please ensure these are updated in your manuscript - after approval of the change(s) - as our production department will not transfer the information in this form to your manuscript.

New acknowledgements:

This study was supported by grants from the National Natural Science Foundation of China (81472800), Key Technology Research and Development Program of Shandong (2019GSF108083), National Natural Science Foundation of China for Young Scientists Fund (82202858), Young Elite Scientists Sponsorship Program by China Association for Science and Technology (2021QNRC001), Cheeloo Young Scholar Project of Shandong University (21320082163154) and project ZR2022QH009 Shandong Provincial Natural Science Foundation.

New Disclosures (financial and non-financial interests, funding):

The authors declare that they have no conflict of interest.

New Author Contributions statement (if applicable per the journal policy):

Benyu Su performed the majority of the laboratory work, the analysis of the data and the writing of the manuscript; Chenyang Qi, Zhongwei Zhang, Junxiao Wang, Fengmei Zhang, David Lim and Chao Dong provided analysis of data, statistical analysis; David Lim provided language editing; Chao Dong and Zhihui Feng were involved in manuscript writing, financial support. Zhihui Feng was involved in conception and design. All authors read and approved the final paper.

State 'Not applicable' if there are no new authors.

SPRINGER NATURE Springer Nature is one of the world's leading global research, educational and professional publishers, created in May 2015 through the combination of Nature Publishing Group, Palgrave Macmillan, Macmillan Education and Springer Science+Business Media.

SPRINGER NATURE

Change of authorship request form - Journals (pre-acceptance)

Section 6: Declaration of agreement. *All* authors, unchanged, new and removed *must* sign this declaration.
(NB: Please print the form, (docu)-sign and return/upload a scanned copy. Please note that signatures that have been inserted as an image file are acceptable as long as it is handwritten. Typed names in the signature box are unacceptable.) *** Please delete as appropriate. Delete all of the bold if you were on the original authorship list and are remaining as an author.**

	First name	Family name		Signature	Date
1st author	Benyu	Su	I agree to the proposed new authorship shown in section 4 /**and the addition/removal*of my name to the authorship list** /and the proposed change in corresponding author	Benyu Su	2 February 2023
2nd author	David	Lim	I agree to the proposed new authorship shown in section 4 /**and the addition/removal*of my name to the authorship list** /and the proposed change in corresponding author	David Lim	2 February 2023
3rd author	Chenyang	Qi	I agree to the proposed new authorship shown in section 4 /**and the addition/removal*of my name to the authorship list** /and the proposed change in corresponding author	Chenyang Qi	2 February 2023
4th authors	Zhongwei	Zhang	I agree to the proposed new authorship shown in section 4 /**and the addition/removal*of my name to the authorship list** /and the proposed change in corresponding author	Zhongwei Zhang	2 February 2023
5th author	Junxiao	Wang	I agree to the proposed new authorship shown in section 4 /**and the addition/removal*of my name to the authorship list** /and the proposed change in corresponding author	Junxiao Wang	2 February 2023
6th author	Fengmei	Zhang	I agree to the proposed new authorship shown in section 4 /**and the addition/removal*of my name to the authorship list** /and the proposed change in corresponding author	Fengmei Zhang	2 February 2023
7th author	Chao	Dong	I agree to the proposed new authorship shown in section 4 /**and the addition/removal*of my name to the authorship list** /and the proposed change in corresponding author	Chao Dong	2 February 2023

SPRINGER NATURE Springer Nature is one of the world's leading global research, educational and professional publishers, created in May 2015 through the combination of Nature Publishing Group, Palgrave Macmillan, Macmillan Education and Springer Science+Business Media.

SPRINGER NATURE

Change of authorship request form - Journals (pre-acceptance)

	First name	Family name		Signature	Date
8th author	Zhihui	Feng	I agree to the proposed new authorship shown in section 4 /**and the addition/removal*of my name to the authorship list** /and the proposed change in corresponding author	Zhihui Feng	2 February 2023
9th author			I agree to the proposed new authorship shown in section 4 /**and the addition/removal*of my name to the authorship list** /and the proposed change in corresponding author		
10th author			I agree to the proposed new authorship shown in section 4 /**and the addition/removal*of my name to the authorship list** /and the proposed change in corresponding author		

Please use an additional sheet if there are more than 10 authors.

In case of author collaborations with formal agreement:

	Name of consortium/consortia	First name	Family name		Signature	Date
Representative/legal guarantor				I agree to the proposed new authorship shown in section 4 /**and the addition/removal*of my name to the authorship list** /and the proposed change in corresponding author		

Both added/removed authors should complete the information in the first table under Section 6.

---- End of form ----

SPRINGER NATURE Springer Nature is one of the world's leading global research, educational and professional publishers, created in May 2015 through the combination of Nature Publishing Group, Palgrave Macmillan, Macmillan Education and Springer Science+Business Media.
